# Efficacy of antiviral therapies for COVID-19: a systematic review of randomized controlled trials

**DOI:** 10.1186/s12879-022-07068-0

**Published:** 2022-01-31

**Authors:** Charan Thej Reddy Vegivinti, Kirk W. Evanson, Hannah Lyons, Izzet Akosman, Averi Barrett, Nicole Hardy, Bernadette Kane, Praneeth Reddy Keesari, Yashwitha Sai Pulakurthi, Erin Sheffels, Prasanth Balasubramanian, Richa Chibbar, Spandana Chittajallu, Kathryn Cowie, J. Karon, Lauren Siegel, Ranita Tarchand, Caleb Zinn, Nitin Gupta, Kevin M. Kallmes, Kavitha Saravu, Jillienne Touchette

**Affiliations:** 1 Department of Internal Medicine, Jacobi Medical Center, Albert Einstein College of Medicine, 1400 Pelham Pkwy S, Bronx, NY USA; 2 Superior Medical Experts, 1425 Minnehaha Ave E, P.O. Box 6000545, St Paul, MN 55106 USA; 3 Nested Knowledge, 1430 Avon Street N, Saint Paul, MN 55117 USA; 4 Ohio University Heritage College of Osteopathic Medicine, 6775 Bobcat Way, Dublin, OH 43016 USA; 5 Kamineni Academy of Medical Sciences and Research Center, Hyderabad, Telangana 500068 India; 6 Department of Medicine, Lakeridge Health, 1 Hospital Crt, Oshawa, ON L1G 2B9 Canada; 7 Reading Hospital, 420 South 5th Avenue, West Reading, PA 19611 USA; 8 Department of Infectious Disease, Kasturba Medical College, Manipal, Manipal Academy of Higher Education, Manipal, Karnataka 576104 India; 9 Manipal Center for Infectious Diseases, Prasanna School of Public Health, Manipal Academy of Higher Education, Manipal, Karnataka 576104 India; 10 Weill Cornell Medical College, 1300 York Ave, New York, NY 10065 USA

**Keywords:** Systematic review, COVID-19, Antiviral, SARS-CoV-2, Therapeutic, Randomized controlled trial

## Abstract

**Background:**

Coronavirus disease 2019 (COVID-19) continues to pose a significant threat to public health worldwide. The purpose of this study was to review current evidence obtained from randomized clinical trials on the efficacy of antivirals for COVID-19 treatment.

**Methods:**

A systematic literature search was performed using PubMed to identify randomized controlled trials published up to September 4, 2021 that examined the efficacy of antivirals for COVID-19 treatment. Studies that were not randomized controlled trials or that did not include treatment of COVID-19 with approved antivirals were excluded. Risk of bias was assessed using the Scottish Intercollegiate Guidelines Network (SIGN) method. Due to study heterogeneity, inferential statistics were not performed and data were expressed as descriptive statistics.

**Results:**

Of the 2,284 articles retrieved, 31 (12,440 patients) articles were included. Overall, antivirals were more effective when administered early in the disease course. No antiviral treatment demonstrated efficacy at reducing COVID-19 mortality. Sofosbuvir/daclatasvir results suggested clinical improvement, although statistical power was low. Remdesivir exhibited efficacy in reducing time to recovery, but results were inconsistent across trials.

**Conclusions:**

Although select antivirals have exhibited efficacy to improve clinical outcomes in COVID-19 patients, none demonstrated efficacy in reducing mortality. Larger RCTs are needed to conclusively establish efficacy.

**Supplementary Information:**

The online version contains supplementary material available at 10.1186/s12879-022-07068-0.

## Background

Coronavirus disease 2019 (COVID-19) continues to present a significant challenge to healthcare systems worldwide, with approximately 269 million confirmed cases of the disease that have led to 5.3 million deaths as of December 12, 2021 [1]. COVID-19 develops from a viral infection, severe acute respiratory syndrome coronavirus 2 (SARS-CoV-2), which can elicit exaggerated immune and inflammatory responses if the infection progresses [2]. As such, there are a wide variety of therapeutic strategies that have been used to treat the disease at various stages, including antiviral, antiretroviral, antimalarial, anti-inflammatory, corticosteroid, immunomodulatory, and immunoglobulin therapies [3].

Research on drug therapies for COVID-19 has relied heavily on results obtained from observational studies, many of which contain biases resulting from demographical differences, patient/disease heterogeneity, differences in institutional practices and standards, and differences in healthcare infrastructure and financial support. As a result of the substantial heterogeneity across studies, a consensus on COVID-19 therapies has remained elusive.

Antiviral drugs, such as remdesivir, represent promising drug candidates to attenuate viral and disease progression. Although there have been comprehensive presentations of outcomes associated with antiviral treatments for COVID-19 obtained from randomized controlled design, the number of relevant randomized controlled trials were limited in these studies because they were either published early in the pandemic [4] or had search dates that ended during the middle of the pandemic [5] and many new trails have been published in the past year. Additionally, while a more recent review has been published, it did not include a description of how the study was carried out and was not PRISMA compliant [6]. Here, we conducted a systematic review of RCTs that examined antiviral efficacy for COVID-19 treatment.

## Methods

### Literature search

A systematic literature search was conducted to identify RCTs that investigated antiviral treatments of COVID-19 using PubMed through Nested Knowledge, an AutoLit platform for living systematic reviews [7]. The search terms used are listed in Table 1, and search filters or limits were not used. All fields were searched and the search was not limited to title/abstract. Databases used included Embase, PubMed, PubMed Central, and Web of Science. This study was conducted in accordance with the Preferred Reporting Items for Systematic Reviews and Meta-Analyses (PRISMA) guidelines [8]. A review protocol was created by the authors in order to establish the framework for this systematic review and can be viewed on the Nested Knowledge platform [9]. Concepts outlined in the protocol were then developed into a custom tagging hierarchy in order to tag each study, which reflected specific evidence underneath the categories we laid out. For example, under outcomes, there is a node for Clinical Improvement that reflects an outcome we intended to gather from each study. Tagging of full-text articles was completed in order to trace concepts and link qualitative synthesis. The review was not registered.

**Table 1:** Search terms

Search terms	Database	Search date	Number of results
(Lopinavir OR Ritonavir OR Remdesivir OR Ribavirin OR Arbidol OR Favipiravir OR Sofosbuvir OR Daclatasvir) AND (COVID-19 OR SARS-COV-2 OR "novel coronavirus") AND (RCT OR "randomized controlled trial" OR "randomised controlled trial" OR "randomized" OR "randomised")	Web of Science	12-01-2021	336
(Lopinavir OR Ritonavir OR Remdesivir OR Ribavirin OR Arbidol OR Favipiravir OR Sofosbuvir OR Daclatasvir) AND (COVID-19 OR SARS-COV-2 OR "novel coronavirus") AND (RCT OR "randomized controlled trial" OR "randomised controlled trial" OR "randomized" OR "randomised")	Embase	12-01-2021	25
(Lopinavir OR Ritonavir OR Remdesivir OR Ribavirin OR Arbidol OR Favipiravir OR Sofosbuvir OR Daclatasvir) AND (COVID-19 OR SARS-COV-2 OR "novel coronavirus") AND (RCT OR "randomized controlled trial" OR "randomised controlled trial" OR "randomized" OR "randomised")	PubMed	12-01-2021	339
(Lopinavir OR Ritonavir OR Remdesivir OR Ribavirin OR Arbidol OR Favipiravir OR Sofosbuvir OR Daclatasvir OR Ivermectin OR Azithromycin) AND (COVID-19 OR SARS-COV-2 OR "novel coronavirus") AND (RCT OR "randomized controlled trial")	PubMed	12-04-2021	162
("Therapeutics" OR "antiviral therapies") AND (RCT OR "randomized controlled trial") AND (COVID-19 OR SAR-COV-2 OR "coronavirus")	PubMed	1-04-2021	47
("randomized controlled trial" OR RCT) AND (Ribavirin) AND (COVID-19 OR SARs-CoV-2 OR "coronavirus" OR SAR-COV-2)	PubMed	12-04-2021	14
(SARs-CoV-2 OR SARs OR COVID-19 OR "coronavirus") AND (LPV/RTV OR Lopinavir OR Ritonavir) AND (RCT or "randomized controlled trial")	PubMed	12-05-2021	68
(Sofosbuvir OR Daclatasvir) AND (RCT OR "randomized controlled trial") AND (COVID-19 OR SAR-COV-2 OR "novel coronavirus")	PubMed	12-04-2021	7
SARs-CoV-2 OR SARs OR COVID-19 OR "coronavirus" OR covid AND ("antiviral drugs") AND (RCT OR "randomized controlled trial" OR "randomised controlled trial")	PubMed	12-04-2021	14
("antiviral therapies" OR "antiviral drugs") AND (RCT OR "randomized controlled trial" OR "randomised controlled trial") AND (COVID-19 OR SAR-COV-2 OR coronavirus OR covid)	PubMed	12-04-2021	20
(SARs-CoV-2 OR SARs OR COVID-19 OR "coronavirus" OR "covid") AND (LPV/RTV OR Lopinavir OR Ritonavir) AND ("randomised controlled trial" OR RCT or "randomized controlled trial")	PubMed	12-04-2021	73
(Lopinavir OR Ritonavir OR Remdesivir OR Ribavirin OR Arbidol OR Favipiravir OR Sofosbuvir OR Daclatasvir) AND (COVID-19 OR SARS-COV-2 OR "novel coronavirus" OR covid) AND (RCT OR "randomized controlled trial" OR "randomised controlled trial"))	PubMed	12-04-2021	124
("novel coronavirus" OR COVID-19 OR SARS-CoV-2) AND (RCT OR "randomized controlled trial") AND ("antiviral therapy") AND (Lopinavir OR Ritonavir OR Remdesivir OR Ribaviron OR Arbidol OR Favipiravir OR Daclatasvir OR Sofosbuvir)	PubMed	12-04-2021	11
(Lopinavir OR Ritonavir OR Remdesivir OR Ribavirin OR Arbidol OR Favipiravir OR Sofosbuvir OR Daclatasvir) AND (COVID-19 OR SARS-COV-2 OR "novel coronavirus") AND (RCT OR "randomized controlled trial")	PubMed Central	02-08-2021	1971
(Lopinavir OR Ritonavir OR Remdesivir OR Ribavirin OR Arbidol OR Favipiravir OR Sofosbuvir OR Daclatasvir) AND (COVID-19 OR SARS-COV-2 OR "novel coronavirus") AND (RCT OR "randomized controlled trial")	PubMed	12-04-2021	124
("COVID-19" OR "coronavirus" OR SAR-COV-2) AND ("Ribavirin") AND (RCT OR "randomized control trial")	PubMed	12-04-2021	5

### Study selection and quality assessment

Studies published between November 1, 2019 and September 4, 2021 were considered. Prior to screening, all studies published before November 1, 2019 or not published in English were automatically excluded by Nested Knowledge. Additionally, during the screening process, a machine learning algorithm ordered studies based on what was most likely to be included, and the software automatically de-duplicated studies. No further automation was used, as each article was screened by one of nine contributors and inclusion was independently verified by one author (NH). All studies that used a randomized controlled design to examine clinical outcomes related to antiviral treatment of COVID-19 were included. Only drugs approved for use as antivirals were considered, including baloxavir marboxil [10], lopinavir/ritonavir (LPV/r) [11], atazanavir [12], sofosbuvir [13], daclatasvir [14], remdesivir [15], ribavirin [16], favipiravir [17], umifenovir (Arbidol) [18], and azvudine [19] and novaferon [20]. The following article types were excluded: observational, editorial, opinion, in vitro or in vivo study, review, methods, case series or report, guidelines, and articles that were not published in English.

### Data collection

Data was manually extracted through the Nested Knowledge platform for living systematic reviews by one of 11 contributors and independently checked for accuracy by one author for each study. Tags from the custom-made Nested Knowledge tagging hierarchy were pre-configured as data elements in order to keep variables organized. Variables in the platform were classified as continuous, categorical, or dichotomous, and manually extracting data from full-text articles facilitated statistical analysis and qualitative synthesis. When available, background characteristics were collected, including age, sex, time from symptom onset to the start of treatment, white blood cell count (WBC), and oxygen saturation (SpO_2_). Intervention-related information, such as doses and regiment, follow-up period, and concomitant medications, were also collected. The outcomes collected included mortality, incidence of mechanical ventilation and intensive care unit (ICU) admission, number of patients with negative reverse transcription polymerase chain reaction (RT-PCR) tests, duration of hospitalization, incidence of clinical improvement, and improvement in SpO_2_.

### Risk of bias and statistical analysis

Risk of bias was assessed using the Scottish Intercollegiate Guidelines Network (SIGN) checklist for randomized controlled trials [21]. Items that are considered in the SIGN checklist include an appropriate and clearly focused question, randomized assignment, adequate concealment, blinding, similar treatment and control groups at the start of the trial, the treatment is the only difference between groups, standard outcome measurement, percentage of subjects that dropped, intention to treat analysis, comparable results for all sites, and overall assessment of the study. The grading system includes levels of evidence rated from 1 +  + high quality to 2- high risk of bias, as well as grades of recommendation, followed by grades of recommendation from grade A to D. Two independent reviewers assessed each study. Assessments were verified and disagreements were adjudicated by a third reviewer. Due to heterogeneity in treatments used and outcomes reported, inferential statistics were not performed, and data were expressed as descriptive statistics only. Continuous data were reported as mean ± standard deviation (SD) or median (interquartile range [IQR]) unless otherwise noted.

## Results

**Fig. 1:**
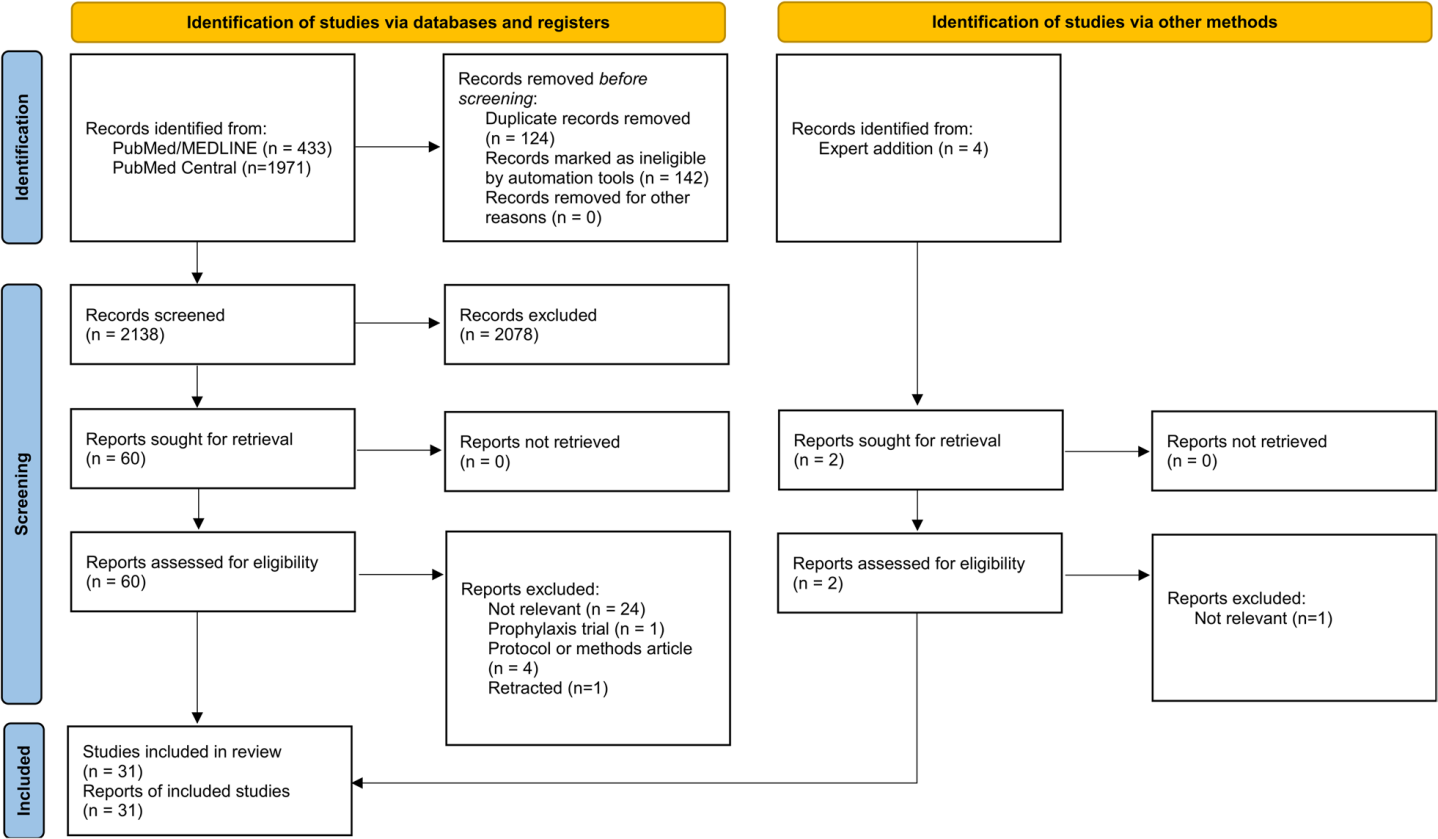
PRISMA flowchart for study inclusion

A total of 2,284 articles were identified from the search terms, of which 31 studies that included 12,440 patients used randomized controlled designs to examine the efficacy of antiviral therapy on COVID-19 [22–53]. A PRISMA diagram detailing the search strategy is shown in Fig. 1. Of the articles identified, 30 were excluded after full-text review [54–83]. One study was originally included, but was later retracted due to concerns about data integrity, and thus was excluded [42]. Antiviral treatments compared in the included studies were umifenovir (Arbidol) [25, 29, 31, 47], baloxavir marboxil [30], enisamium [50], favipiravir [25, 30, 35, 40–42, 44, 45, 48, 52], lopinavir/ritonavir (LPV/r) [24, 26, 27, 29, 31, 37, 38, 44, 47], remdesivir [23, 34, 36, 39, 51, 53], ribavirin [22], sofosbuvir/daclatasvir [22, 32, 33, 46, 49], sofosbuvir/ledipasvir [28], sofosbuvir/ravidasvir [46], and sofosbuvir/velpatasvir [43]. The study characteristics and baseline patient characteristics are summarized in Table 2. The outcomes of interest and study conclusions are summarized in Table 3. Two studies were rated low quality on the risk of bias assessment, with bias favoring the test treatment [49, 51]. The remaining studies were rated either acceptable or high quality (Additional file 1).

**Table 2:** Study and patient characteristics

Author	Study name	Study design	Inclusion criteria	Exclusion criteria	Interventions	Patient characteristics	Patient allocation
Favipiravir							
Bosaeed et al. [40]	Favipiravir and Hydroxychloroquine Combination Therapy in Patients with Moderate to Severe COVID- 19 (FACCT Trial): An Open-Label, Multicenter, Randomized, Controlled Trial	OLRCT	• ≥ 18 years of age •Not pregnant •Diagnosed •with COVID-19 confirmed by RT-PCR •Admitted patients with moderate-to-severe COVID-19 (SaO_2_ of ≤ 94% while breathing ambient air or •significant clinical symptoms with chest x-ray change) •Enrolled within 10 days of disease onset •Written informed consent	•History of myocardial infarction or irregular rhythm/ •QTc in the baseline •ECG of > 490 ms •Comorbidities such as: hematologic malignancy, •advanced (stage 4–5) CKD or dialysis therapy, severe liver damage (Child- •Pugh score ≥ C, AST > five times the upper limit), or HIV	•HCQ + FVP (n = 125) •D1: 1800 mg FVP + 400 mg HCQ, 2x/day •D2-10: 800 mg FVP 2x/day •D2-D5: 200 mg HCQ 2x/day •Also received SoC •SoC (n = 129) •Included other antivirals	•HCQ + FVP (n = 125) •Age, years: 53.03 ± 12.79 •Male: 75 (60%) •WBC, cells/nL 7.77 ± 3.63 •Time Sx onset to Tx start, days: 5.96 ± 2.05 •SoC (n = 129) •Age, years: 52.27 ± 13.36 •Male: 76 (58.91%) •WBC, cells/nL 7.54 ± 3.32 •Time Sx onset to Tx start, days: 5.75 ± 2.07	•HCQ + FVP: •Randomized: 132 •Included in outcome analysis: 125 •SoC: •Randomized:136 •Included in outcome analysis: 129
Chen et al. [25]	Favipiravir versus Arbidol for COVID-19: A Randomized Clinical Trial	OLRCT	• ≥ 18 years of age •Diagnosed as COVID-19 pneumonia according to the Chinese Diagnosis and Treatment Protocol •COVID-19 could be diagnosed without a positive SARS-CoV-2 nucleic acid test result by: (1) a positive chest CT scan; (2) significant clinical manifestation (3) laboratory results indicating lymphopenia •Enrolled within 12 days of initial symptoms •Voluntarily provided informed consent	•Allergic to FVP or ARB •Elevated ALT/AST (> 6 × upper limit of normal range) or with chronic liver disease (cirrhosis at grade Child–Pugh C) •Severe/critical patients whose expected survival time were < 48 h •Pregnant •HIV infection •Considered unsuitable by researcher	•FVP (n = 116) •D1: 1600 mg FVP 2x •D2-7: 600 mg FVP 2x/day •ARB (n = 120) •200 mg ARB 3x/day •7–10 days	•FVP (n = 116) •Age, years: • < 65 years: 87 (75.00%) • ≥ 65 year: 29 (25.00%) •Male: 59 (50.86%) •ARB (n = 120) •Age, years: • < 65 years: 79 (65.83%) • ≥ 65 years: 41 (34.17%) •Male: 51 (42.50%)	•FVP: •Randomized: 120 •Included in outcome analysis: 116 •ARB: •Randomized:120 •Included in outcome analysis: 120
Dabbous et al. [41]	Efficacy of favipiravir in COVID-19 treatment: a multi-center randomized study	OLRCT	•18–80 years of age •Confirmed SARS-CoV-2 infection with mild or moderate symptoms •Hospital admission three days after the symptom onset •Agreed to participate in the study and signed an informed consent	•Allergic or contraindication to the drug •Pregnant or lactating •Cardiac problems •Liver or renal failure •Other organ failure	•FVP (n = 44) •D1: 1600 mg FVP 2x/day •D2-10: 600 mg FVP 2x/day •Also received SoC •CQ (n = 48) •D1-10: CQ 600 mg tablets 2x/day •Also received SoC	•FVP (n = 44) •Age, years: 34.86 ± 15.95 •Male: 20 (45.5%) •WBC, cells/nL 6.58 ± 2.99 •CQ (n = 48) •Age, years: 36.15 ± 17.67 •Male: 25 (52.1%) •WBC, cells/nL 5.60 ± 2.61	•FVP •Randomized: 48 •Included in outcome analysis: 44 •CQ •Randomized: 48 •Included in outcome analysis: 48
Doi et al. [48]	A Prospective, Randomized, Open-Label Trial of Early versus Late Favipiravir Therapy in Hospitalized Patients with COVID-19	OLRCT	• ≥ 16 years of age •Inpatient status •Positive •RT-PCR for SARS-CoV-2 from a pharyngeal or nasopharyngeal swab specimen collected within 14 days •Eastern Cooperative Oncology Group (ECOG) performance status of 0 or 1 (12) •Ability to remain •hospitalized for 6 days or longer •Negative pregnancy test (premenopausal female only) •Written consent for participation	•Performance status of 2 or greater •Severe hepatic disease •Need for dialysis •Altered mental status •Pregnancy •Female patients who did not agree to •use effective contraceptive methods •Male patients with female partners who did not agree to the •use of effective contraceptive methods •Hereditary xanthinuria •Hypouricemia or history of •xanthine urolithiasis •Uncontrolled gout or hyperuricemia •Immunosuppressive conditions •Receipt of systemic antiviral agent against SARS-CoV-2 within 28 days	•Early treatment FVP (n = 44) •D1: 1800 mg FVP 2x/day •D2-10: 800 mg 2x/day •Late treatment FVP (n = 44) •D6: 1800 mg FVP 2x/day FVP •D6-16: 800 mg 2x/day	•Early treatment FVP (n = 44) •Age, years: 48.0 (34.5, 68.0) •Male: 23 (52.3%) •SpO_2_ adm: 96.0% •WBC, cells/nL: 4.4 (3.6, 5.8) •Time Sx onset to Tx start, days: 7.0 (5.5, 10.0) •Late treatment FVP (n = 44) •Age, years: 51.0 (39.5, 62.0) •Male: 31 (70.5%) •SpO_2_ adm: 96.0% •WBC, cells/nL: 5.1 (4.0, 6.4) •Time Sx onset to Tx start, days: 8.0 (5.0, 10.0)	•Early FVP •Randomized: 44 •Included in outcome analysis: 44 •With positive RT-PCR on D1: 36 •Late FVP •Randomized: 45 •Included in outcome analysis: 44 •With positive RT-PCR on D1: 33
Lou et al. [30]	Clinical Outcomes and Plasma Concentrations of Baloxavir Marboxil and Favipiravir in COVID-19 Patients: An Exploratory Randomized, Controlled Trial	OLRCT	• ≥ 18 years of age •voluntarily provided informed consent •Confirmed COVID-19 infection by RT-PCR •No difficulty swallowing oral drugs ••Ability to follow protocol according to the judgment of researchers	•Allergic to any related drugs •Weight < 40 kg •Critical illness (respiratory failure and mechanical ventilation, shock, other organ failure requiring ICU treatment) •Renal insufficiency (estimated creatinine clearance < 60 ml/min) •Abnormal laboratory parameters for: ALT or AST > 5 × ULN, or ALT or AST > 3 × ULN and total bilirubin level > 2 × ULN •Pregnant •BMI > 30 •Considered unsuitable by researcher	•FVP (n = 9) •1600–2200 mg FVP 2 × day 1 •600 mg FVP 3x/day up to 14 days •Control antivirals •B/M (n = 10) •80 mg B/M 1 × day 1,4,7. Day 7 dose for patients with positive test •Control antivirals •Control (n = 10) •100 000 IU IFN-α 3-4x/day •400/100 mg LPV/r 2x/day •800/150 mg D/C 1x/day •200 mg ARB 3x/day	•FVP (n = 9) •Age, years: 58.0 ± 8.1 •Male: 7 (77.8%) •WBC, cells/nL: 7.8 (3.9–14.1) •Time Sx onset to Tx start, days: 8.5 ± 3.7 •B/M (n = 10) •Age, years: 53.3 ± 12.5 •Male: 7 (70.0%) •WBC, cells/nL 8.3 (3.3–27.9) •Time Sx onset to Tx start, days: 12.7 ± 3.5 •Control •Age, years: 46.6 ± 14.1 •Male: 7 (70.0%) •WBC, cells/nL: 6.3 (2.9–19.4) •Time Sx onset to Tx start, days: 13.6 ± 4.6	•No patients excluded from analysis
Shinkai et al. [52]	Efficacy and Safety of Favipiravir in Moderate COVID- 19 Pneumonia Patients without Oxygen Therapy: A Randomized, Phase III Clinical Trial	SBRCT	•20–74 years of age •Positive SARS-CoV-2 based on a nucleic acid amplification test of a respiratory tract sample taken at enrollment •Pulmonary lesions confirmed by chest imaging •Fever > 37.5 °C •Written informed •consent obtained from the patient	• ≥ 11 days since onset of fever of > 37.5 °C •Infection episode was a relapse or reinfection ••SpO_2_ < 94% without oxygen therapy	•FVP (n = 107) •D1: 1800 mg FVP 2x/day •D2-D13: 800 mg 2x/day, duration of treatment variable based on patient improvement •Placebo (n = 49) •Matching placebo tablets for up to ••14 days	•FVP (n = 107) •Age, years: 43.8 ± 12.5 •Male: 76 (71.0%) •SpO2 adm: 96.1 ± 1.7 •Placebo (n = 49) •Age, years: 48.7 ± 14.1 •Male: 28 (57.1%) •SpO2 adm: 96.0 ± 2.1	•No patients excluded from analysis
Solaymani-Dodaran et al. [44]	Safety and efficacy of Favipiravir in moderate to severe SARS-CoV-2 pneumonia	OLRCT	•16–100 years of age •Diagnosis of SARS-CoV-2 based on either a positive RT-PCR test or typical ground •glass appearance on chest CT scan in need of hospital admission due to a SpO_2_ of ≤ 93% •Informed and written consent	•History of receiving any antiviral drug (Ribavirin, •Oseltamivir, and LPV/r) for current illness •History of •chronic renal or liver failure, or gastrointestinal bleeding • < 48 h life expectancy •Pregnant or lactating females •Known HIV infection/AIDS •QT interval > 500 ms in •ECG	•FVP (n = 190) •1600 mg FVP immediately •D1-D7: 600 mg FVP every 8 h + 200 mg HCQ 2x/day •Daily HCQ reduced to a single dose on D1 after trial started •LPV/r (n = 183) •200 mg HCQ on admission D1-D7: 400 mg/100 mg LPV/r 2x/day	•FVP (n = 190) •Age, years: 58.6 ± 17.5 •Male: 115 (60.5%) •SpO_2_ adm: 89 (5) •WBC, cells/nL (n = 184): 6.9 (5.1–8.9) •LPV/r (n = 183) •Age, years: 56.6 ± 17.1 •Male: 90 (49.2%) •SpO2 adm: 89 (7) WBC, cells/nL (n = 169): 6.3 (4.9–9.1)	•FVP •Randomized: 216 •Included in outcome analysis: 190 •LPV/r •Randomized: 208 •Included in outcome analysis: 183
Udwadia et al. [35]	Efficacy and safety of favipiravir, an oral RNA-dependent RNA polymerase inhibitor, in mild-to-moderate COVID-19: A randomized, comparative, open-label, multicenter, phase 3 clinical trial	OLRCT	•18–75 years of age •Patients admitted to the hospital with mild (including asymptomatic) to moderate COVID-19 •Confirmed SARS-CoV-2 virus by RT-PCR within 48 h prior to randomization •No participation in any other interventional clinical study •Agreement to use effective contraception during study and for ≥ 7 days following the last treatment •Negative pretreatment pregnancy test for female patients of child-bearing potential ••Time from symptom onset to randomization no more than 7 days for mild disease and 10 days for moderate disease	•Severe infection •SpO_2_ ≤ 93% or PaO2/FiO_2_ ≤ 300 mmHg •Current ICU care for the management of ongoing clinical status •Inability to take or tolerate oral medications •Allergy or hypersensitivity to favipiravir •Asthma or chronic obstructive lung disease •Severe liver disease •History of gout or hyperuricemia •Prolonged QT (QTc ≥ 450 ms for men and as QTc ≥ 470 ms for women) •Severely reduced left ventricular function •Severe renal impairment or having received continuous renal replacement therapy, hemodialysis or peritoneal dialysis •Prohibited concomitant medications included: HCQ or CQ, pyrazinamide, repaglinide, theophylline and famciclovir or sulindac	•FVP (n = 72) •D1: 1800 mg favipiravir 2x/day •D2-D14: 800 mg favipiravir 2x/day + standard supportive care •Control (n = 75) •Supportive care alone	•FVP (n = 72) •Age, years: 43.6 ± 12.2 •Male: 51 (70.8%) •Control (n = 75) •Age, years: 43.0 ± 11.2 •Male: 57 (76.0%)	•FVP •Randomized: 75 •Included in outcome analysis: 72 •Control •Randomized: 75 •Included in outcome analysis: 75
Zhao et al. [45]	Favipiravir in the treatment of patients with SARS-CoV-2 RNA recurrent positive after discharge: A multicenter, open-label, randomized trial	OLRCT	• ≥ 18 years of age •After first diagnosis and treatment of COVID-19, 2 consecutive (24 h apart) negative SARS-CoV-2 RNA tests of sputum or nasopharyngeal swabs •During screening (follow-up after discharge), a positive SARS-CoV-2 RNA tests of sputum, nasopharyngeal swabs, blood, feces, or other specimen •Volunteered to participate in the research	•FVP allergy •Pregnant or lactating ••Patient determined unsuitable for participation	•FVP (n = 36) •1600 mg 2 × day 1 •600 mg 2 × day 2–7 •As needed after day 7 until day 14 •Control (n = 19) •Received drugs other than favipiravir and treatment according to the needs of the disease	•FVP •Age, years: 55.8 ± 14.2 •Male: 16 (44.4%) •WBC, cells/nL 5.9 ± 1.8 •Control •Age: 55.5 ± 12.6 •Male: 9 (47.4%) •WBC, cells/nL 5.7 ± 1.4	•No patients excluded from analysis
•Lopinavir/Ritonavir							
Ader et al. [37]	An open-label randomized, controlled trial of the effect of lopinavir/ritonavir, lopinavir/ ritonavir plus IFN-β-1a and hydroxychloroquine in hospitalized patients with COVID-19	OLRCT	• ≥ 18 years of age •Laboratory-confirmed SARS-CoV-2 infection by PCR, or other commercial or public health assay in any specimen < 72 h prior to randomization •Hospitalized patients with illness of any duration, and at least one of the following: oClinical assessment (evidence of rales/crackles on physical examination) AND SpO_2_ ≤ 94% on room air, OR oacute respiratory failure requiring supplemental oxygen, high flow oxygen devices, non-invasive ventilation, and/or mechanical ventilation •Women of childbearing potential must agree to use contraception for the duration of the study	•Refusal to participate expressed by patient or legally authorized representative if they are present •Spontaneous blood alanine transferase (ALT)/AST levels > 5 times the upper limit of normal •Stage 4 severe CKD or requiring dialysis •Pregnancy or breast-feeding •Anticipated transfer to another hospital, which is not a study site within 72 h •Patients previously treated with one of the antivirals evaluated in the trial in the past 29 days •Contraindication to any study medication including allergy •Use of medications that are contraindicated with LPV/r and HCQ •HIV infection under HAART •History of severe depression or attempted suicide or current suicidal ideation •cQT interval > 500 ms	•LPV/r (n = 145) •D1-D14: 400 mg/100 mg LPV/r 2x/day •SoC •LPV/r + IFN (n = 145) •D1-D14: 400 mg/100 mg LPV/r 2x/day •D1, D3, D6: 44 μg of •subcutaneous IFN-β-1a •SoC •HCQ (n = 145) •D1: 400 mg HCQ 2x/day •D2-D9: 400 mg HCQ 1x/day •SoC •Control (n = 148) •SoC	•LPV/r (n = 145) •Age, years: 63 (55–71) •Male: 106 (73.1%) •Time Sx onset to Tx start, days: 10.0 (7.0–13.0) •LPV/r + IFN (n = 145) •Age, years: 64 (53–71) •Male: 103 (71.0%) •Time Sx onset to Tx start, days: 10.0 (7.0–12.0) •HCQ (n = 145) •Age, years: 65 (55–71) •Male: 104 (71.7%) •Time Sx onset to Tx start, days: 8.0 (7.0–11.0) •Control (n = 148) •Age, years: 62 (52–71) •Male: 105 (70.9%) •Time Sx onset to Tx start, days: 9.0 (7.0–12.0)	•Total patients •Randomized: 603 •Included in outcome analysis: 583 •LPV/r •Included in outcome analysis: 145 •LPV/r + IFN •Included in outcome analysis: 145 •HCQ •Included in outcome analysis: 145 •Control •Included in outcome analysis: 148
Alavi Darazam et al. [47]	Umifenovir in hospitalized moderate to severe COVID-19 patients: A randomized clinical trial	OLRCT	• ≥ 18 years of age •Presence of at least one of the following manifestation: (radiation contactless body temperature ≥ 37.5 ◦C, cough, shortness of breath, nasal congestion/discharge, •myalgia/arthralgia, diarrhea/vomiting, headache or fatigue) •Peripheral capillary SpO_2_ ≤ 93% on pulse oximetry •Respiratory frequency ≥ 24/minute while breathing ambient air (on admission day) •Acute onset of symptoms ≤ 14 days	•Consumption of potentially interacting •medications with LPV/r or IFN-β-1a •Pregnancy and •breastfeeding •History of alcohol use disorder, or any illicit drug dependence within the past five years •Blood AST/ALT levels ≥ fivefold higher relative to maximum limit of normal range on laboratory findings •Participation refusal who needed invasive ventilation from the beginning	•LPV/r + HCQ + IFN-β-1a + ARB (n = 51) •LPV/r (400 mg/100 mg bid for 10–14 •days) + HCQ (400 mg single dose) + interferon-β1a (Subcutaneous injections of 44 μg (12,000 IU) on days 1, 3, 5) + ARB (200 mg trice daily for 10 days) •Control (n = 50) •LPV/r (same dose) + HCQ (same dose) + IFN-β-1a (same dose)	•LPV/r + HCQ + interferon-β-1a + ARB (n = 51) •Age, years: 62.1 ± 15.3 •Male: 31 (60.8%) •SpO_2_: 85 (80–85) •WBC, cells/nL • < 4: 8 (18.2%) •4-10: 33 (75.0%) • > 10: 3 (6.8%) •Control (n = 50) •Age, years: 60.2 ± 16.5 •Male: 26 (52.0%) •SpO_2_: 86 (80–88) •WBC, cells/nL o < 4: 10 (21.7%) o4-10: 29 (63.0%) o > 10: 7 (15.2%)	•No patients excluded from analysis
Arabi et al. [38]	Lopinavir-ritonavir and hydroxychloroquine for critically ill patients with COVID-19: REMAP-CAP randomized controlled trial	OLRCT	•Adults ≥ 18 years of age •Admitted with suspected or confirmed COVID-19 •Receiving respiratory or cardiovascular organ failure support •in an ICU •In addition to patients enrolled in the COVID-19 Antiviral Therapy •Domain, the primary model included patients enrolled in other domains in Severe State, for covariate adjustment	•Death deemed to be imminent during the next 24 h AND one or more of the patient, substitute decision-maker, or attending physician are not committed to full active treatment •Expected discharged from the hospital the same day or the following day •14 + days have elapsed while admitted to hospital with symptoms of an acute illness due to suspected or proven pandemic infection •Previous participation in this REMAP-CAP within last 90 days •Known hypersensitivity to lopinavir-ritonavir and HCQ •Receiving lopinavir-ritonavir or HCQ as a usual medication prior to this hospitalization •Known HIV infection •Severe liver failure •Known or suspected pregnancy •Receiving •amiodarone as a usual medication prior to this hospitalization or any administration of amiodarone within the 72 h prior to the assessment of eligibility •High clinical risk •of sustained ventricular dysrhythmia	•LPV/r (n = 225) •400 mg of lopinavir and 100 mg of ritonavir every 12 h •Administered for 5 days minimum, up to a maximum of 14 days or until ICU •discharge whichever occurred first •HCQ (n = 50) •Two loading •doses of 800 mg, 6 h apart, followed 6 h later by 400 mg •12 hourly for 12 doses •Combination therapy (n = 27) •Control (n = 362)	•LPV/r (n = 225) •Age, years: 61.0 ± 13.0 •Male: 182 (71.7%) •HCQ (n = 50) •Age, years: 56.3 ± 13.0 •Male: 35 (70%) •Combination therapy (n = 27) •Age, years: 60.3 ± 8.9 •Male: 19 (70.4%) •Control (n = 362) •Age, years: 60.8 ± 12.9 •Male: 252 (69.6%)	•LPV/r: •Randomized: 268 •Included in analysis: 249 •HCQ: •Randomized: 52 •Included in analysis: 49 •Combination therapy: •Randomized: 29 •Included in analysis: 26 •Control: •Randomized: 377 •Included in analysis: 353
Cao et al. [24]	A Trial of Lopinavir–Ritonavir in Adults Hospitalized with Severe Covid-19	OLRCT	•Positive RT-PCR •Male and nonpregnant female patients ≥ 18 years of age •Pneumonia confirmed by chest imaging •Sao_2_ ≤ 94% while breathing ambient air or a ratio of Pao_2_:Fio_2_ ≤ 300 mg Hg	•Physician decision that involvement in the trial was not in the patient’s best interest •Any condition that would not allow the protocol to be followed safely •Known allergy or hypersensitivity to lopinavir–ritonavir •Known severe liver disease (e.g., cirrhosis, ALT > 5 × upper limit of the normal range or AST > 5 × upper limit of the normal range) •Use of medications that are contra-indicated with lopinavir–ritonavir and that could not be replaced or stopped during the trial period •Pregnancy or breastfeeding •Known HIV infection	•LPV/r •D1-D14: 400 mg/100 mg LPV/r 2x/day •SoC •Control •SoC	•LPV/r (n = 99) •Age, years: 58.0 (50.0, 68.0) •Male: 61 (61.6%) •WBC, cells/nL: 7.3 (5.3, 9.6) •Time Sx onset to Tx start, days: 13 (11, 17) •Control (n = 100) •Age, years: 58.0 (48.0, 68.0) •Male: 59 (59.0%) •WBC, cells/nL 6.9 (4.9, 9.1) •Time Sx onset to Tx start, days: 3 (10, 16)	•LPV/r: •Randomized: 99 •Included in analysis: 96 •Control: •Randomized: 100 •Included in analysis: 100
Li et al. [29]	Efficacy and safety of lopinavir/ritonavir or arbidol in adult patients with mild/moderate COVID-19: an exploratory randomized controlled trial	SBRCT	•18–80 years of age •SARS-CoV-2 confirmed by RT-PCR from pharyngeal swab •Mild clinical status “defined as having mild clinical symptoms but no signs of pneumonia on imaging” OR moderate clinical status “defined as having fever, respiratory symptoms and pneumonia on imaging” •Lab findings: (1) creatinine ≤ 110 μmol/L, (2) creatinine clearance rate (eGFR) ≥ 60 ml/min/1.73m^2^, (3) aspartate aminotransferase (AST) and alanine aminotransferase (ALT) ≤ 5 × ULN, and (4) total bilirubin (TBIL) ≤ 2 × ULN •Voluntarily and provided informed consent	•Known or suspected to be allergic to LPV/r or ARB •Severe nausea, vomiting, diarrhea, or other complaints affecting oral intake or absorption in the digestive tract •Medications contraindicated with LPV/r or ARB •Serious underlying diseases (including but not limited to heart, lung, or kidney disease, liver malfunction, or mental illnesses affecting treatment compliance) •Complications with pancreatitis or hemophilia prior to the trial •Pregnant or lactating females •Suspected or confirmed history of alcohol or substance use disorder •Participation in other drug trials within the past month •Considered unsuitable by researchers	•LPV/r (n = 34) •D1-D14: 500 mg/100 mg LPV/r 2x/day •Minimum 7 days treatment •ARB (n = 35) •D1-D14: 200 mg 3x/day •Minimum 7 days •Control (n = 17) •Supportive care only	•LPV/r (n = 34) •Age, years: 50.7 ± 15.4 •Male: 17 (50.0%) •WBC, cells/nL o < 4: 8 (23.5%) o4-10: 25 (73.5%) o > 10: 1 (2.9%) •Time Sx onset to Tx start, days: 3.5 (2, 6) •ARB (n = 35) •Age, years: 50.5 ± 14.6 •Male: 16 (45.7%) •WBC, cells/nL • < 4: 11 (31.4%) •4-10: 24 (68.6%) • > 10: 0 (0.0%) •Time Sx onset to Tx start, days: 6 (2, 8) •Control (n = 17) •Age, years: 44.3 (27–62) •Male: 7 (41.2%) •WBC, cells/nL o < 4: 3 (17.6%) o4-10: 14 (82.4%) o > 10: 0 (0.0%) •Time Sx onset to Tx start, days: 5 (2.8%)	•No patients excluded from analysis
Nojomi et al. [31]	Effect of Arbidol (Umifenovir) on COVID-19: a randomized controlled trial	OLRCT	• ≥ 18 years of age •Hospitalized at study center	•Allergy to ARB class of drugs •Abnormal liver or renal function •Abnormal blood coagulation •Pregnant or nursing •Severe heart disease	•LPV/r (n = 50) •D1: 400 mg HCQ 2x/day •D1 + : 400 mg/100 mg LPV/r 2x/day •7–14 days depending on disease severity •ARB (n = 50) •D1: 400 mg HCQ 2x/day •D1 + : 200 mg ARB 3x/day ••7–14 days depending on disease severity	•LPV/r (n = 50) •Age, years: 56.2 ± 14.8 •Male: 27 (54.0%) •SpO2 adm: 84.3% ± 7.7 •WBC: 9.8 ± 5.5 •ARB (n = 50) •Age, years: 56.6 ± 17.8 •Male: 33 (66.0%) •SpO2 adm: 85.5% ± 8.4 ••WBC: 10.5 ± 4.1	•No patients excluded from analysis
RECOVERY collaborative group [26]	Lopinavir–ritonavir in patients admitted to hospital with COVID-19 (RECOVERY): a randomised, controlled, open-label, platform trial	OLRCT	•Admitted patients with clinically suspected or laboratory confirmed SARS-CoV-2 infection •No medical history that might put the patient at substantial risk if they were to participate in the trial •Initially, recruitment limited to patients who were ≥ 18 years of age but from May 9, 2020, this age limit was removed •Written informed consent from all patients or their legal representative	•Patient with severe hepatic insufficiency ••Patients using medicinal products that are highly dependent on cytochrome P450 3A4 for clearance and for whom elevated plasma concentrations would be associated with serious or life-threatening events (in line with the summary of product characteristics)	•LPV/r (n = 1616) •400 mg/100 mg LPV/r 2x/day •maximum 10 days or until discharge, if sooner •Standard care (n = 3424)	•LPV/r (n = 1616) •Age, years: 66.0 ± 16.0 •Male: 973 (60%) •Time Sx onset to Tx start, days: 8 (5, 12) •Standard care (n = 3424) •Age, years: 66.4 ± 15.8 •Male: 2104 (61%) ••Time Sx onset to Tx start, days: 8 (4, 12)	•No patients excluded from analysis
Reis et al. [27]	Effect of Early Treatment With Hydroxychloroquine or Lopinavir and Ritonavir on Risk of Hospitalization Among Patients With COVID-19 The TOGETHER Randomized Clinical Trial	DBRCT	•Adults ≥ 18 years of age • < 8 days since onset of flulike symptoms or chest computerized tomography scan consistent with COVID-19 •At least one additional criterion for high risk: o ≥ 50 years of age oPresence of pulmonary disease oDiabetes requiring oral medication or insulin oHypertension requiring treatment oKnown cardiovascular diseases oSymptomatic lung disease on chronic treatment oHistory of transplantation oObesity oImmunocompromised status due to disease oImmunocompromised status due to medication oPatients with cancer	•Use of any of study drugs in 30 days prior to screening •Clinical •evidence of progression of COVID-19 (i.e., use of oxygen supplementation; arterial oxygen saturation •less than 94%; use of noninvasive positive-pressure ventilation support) •History of known life-threatening cardiac arrhythmias •Long QT syndrome ••Known allergy to study drugs	•LPV/r (n = 244) •D1: 800 mg/200 mg LPV/r 2x/day •D2-D10: 400 mg/100 mg LPV/r 2x/day •HCQ (n = 214) •D1: 800 mg HCQ •D2-D10: 400 mg HCQ •Placebo (n = 227) •Received corresponding tablets of inert material •(talc) •Placebo bottles were matched for the same number of tablets as active HCQ •(placebo of HCQ) and active LPV/r (placebo of LPV/r)	•LPV/r (n = 244) •Age, years: 54 (range: 18–94) •Male: 110 (45.1%) •Time Sx onset to Tx start, days: • > 5: 210 (86.1%) • ≤ 5: 34 (13.9%) •HCQ (n = 214) •Age, years: 53 (range: 18–81) •Male: 92 (43.0%) •Time Sx onset to Tx start, days: o > 5: 177 (82.7%) o ≤ 5: 37 (17.3%) •Placebo (n = 227) •Age, years: 53 (range: 18–80) •Male: 106 (46.7%) •Time Sx onset to Tx start, days: o > 5: 187 (82.4%) o ≤ 5: 40 (17.6%)	•No patients excluded from analysis
**Remdesivir**							
Barratt-Due et al. [39]	Evaluation of the Effects of Remdesivir and Hydroxychloroquine on Viral Clearance in COVID-19: A Randomized Trial	OLRCT	• ≥ 18 years of age •SARS-CoV-2 infection confirmed by RT-PCR •Admitted to the hospital ward or ICU with no anticipated transfer to a non- •study hospital within 72 h of inclusion •Informed consent	•Severe comorbid conditions with life expectancy less than 3 months •Level of AST or ALT > 5 × the upper limit of normal •Rate-corrected QT •interval greater than 470 ms •Pregnancy/ breastfeeding •Acute occurrence of a comorbid condition in a 7-day period before inclusion •Known intolerance to study drugs •Participation in a potentially confounding trial, or concomitant medications interfering with the study drugs	RDV (n = 42) •D1: 200 mg of intravenous RDV •D2 + : 100 mg RDV up to 9 days •SoC RDV control (n = 57) •SoC HCQ (n = 52) •D1: 800 mg of oral •HCQ 2x/ day 1, •D2 + : 400 mg 2x/day, up to 9 days •SoC HCQ control (n = 54) •SoC	RDV (n = 42) •Age, years: 59.7 ± 16.5 •Male: 29 (69.0%) RDV control (n = 57) •Age, years: 58.1 ± 15.7 •Male: 43 (75.4%) HCQ (n = 52) •Age, years: 60.3 ± 13.3 •Male: 31 (59.6%) HCQ control (n = 54) •Age, years: 59.2 ± 16.4 •Male: 34 (63.0%)	RDV: •Randomized: 43 •Included in analysis: 42 RDV Control: •Randomized: 58 •Included in analysis: 57 HCQ: •Randomized: 54 •Included in analysis: 52 HCQ Control: •Randomized: 54 •Included in analysis: 54
Beigel et al. [23]	Remdesivir for the Treatment of Covid-19—Final Report	DBRCT	• ≥ 18 years of age •Admitted with symptoms suggestive of COVID-19 •Provides informed consent •Understands and agrees to comply with planned study procedures •Not pregnant •Laboratory-confirmed SARS-CoV-2 infection (RT-PCR positive in sample collected < 72 h prior to randomization; OR RT-PCR positive in sample collected ≥ 72 h prior to randomization, documented inability to obtain a repeat sample (e.g. due to lack of testing supplies, limited testing capacity, results taking > 24 h, etc.) AND progressive disease suggestive of ongoing SARS-CoV-2 infection •Illness of any duration, and at least one of the following: radiographic infiltrates by imaging, OR SpO_2_ ≤ 94% on room air, OR requiring supplemental oxygen, OR requiring mechanical ventilation •Women of childbearing potential must agree to either abstinence or use at least one primary form of contraception not including hormonal contraception from the time of screening through Day 29 •Agrees to not participate in another clinical trial for the treatment of COVID-19 or SARS-CoV-2 through Day 29	•ALT or AST > 5 × upper limit of normal •eGFR < 30 ml/min (including patients receiving hemodialysis or hemofiltration) •Pregnancy or breast feeding •Anticipated discharge from the hospital or transfer to another hospital which is not a study site within 72 h •Allergy to any study medication	RDV (n = 541) •D1: 200 mg RDV •D2-D10: 100 mg RDV Control (n = 521) •Equal volume of placebo using same schedule as RDV	RDV (n = 541) •Age, years: 58.6 ± 14.6 •Male: 352 (65.1%) •Time Sx onset to Tx start, days: 9 (6, 12) Control (n = 521) •Age, years: 59.2 ± 15.4 •Male: 332 (63.7%) •Time Sx onset to Tx start, days: 9 (7, 13)	RDV: •Randomized: 541 •Included in analysis: 531 Control: •Randomized: 521 •Included in analysis: 517
Goldman et al. [53]	Remdesivir for 5 or 10 Days in Patients with Severe Covid-19	OLRCT	• ≥ 12 years of age •Hospitalized •SARS-CoV-2 infection confirmed by polymerase-chain-reaction assay within 4 days before randomization •Radiographic evidence of pulmonary infiltrates and either had SpO_2_ ≤ 94% while breathing ambient air or were receiving supplemental oxygen	•Patients receiving mechanical ventilation and ECMO at screening •Patients with signs of multiorgan failure •ALT or AST levels > 5 × upper limit of the normal range or estimated creatinine clearance < 50 ml per minute (by the Cockcroft–Gault formula) •Patients receiving concurrent treatment (within 24 h before the start of trial treatment) with other agents with putative activity against Covid-19	5-day RDV (n = 200) •D1: 200 mg RDV •D2-D5: 100 mg RDV 10-day RDV (n = 197) •D1: 200 mg RDV •D2-D9: 100 mg RDV	5-day RDV (n = 200) •Age, years: 61 (50, 69) •Male: 120 (60.0%) •Time Sx onset to Tx start, days: 8 (5, 11) 10-day RDV (n = 197) •Age, years: 62 (50, 71) •Male: 133 (67.5%) •Time Sx onset to Tx start, days: 9 (6, 12)	No patients excluded from analysis
Mahajan et al. [51]	Clinical outcomes of using remdesivir in patients with moderate to severe COVID-19: A prospective randomised study	OLRCT	•18–60 years of age •Hospitalized •SARS-CoV-2 infection confirmed by RT-PCR within the last 4 days •Radiographic evidence of pneumonia •Respiratory rate > 24/min •Oxygen saturation of ≤ 94% •Creatinine clearance > 40 ml per minute	•Patients receiving mechanical ventilation •Patients with multi organ failure •ALT and AST levels greater than 3 × ULN	RDV + SoC (n = 34) •D1: 200 mg RDV •D2-D5: 100 mg RDV SoC (n = 36)	RDV + SoC (n = 34) •Age, years: 58.1 ± 12.1 •Male: 21 (61.7%) •Time Sx onset to Tx start, days: 6.26 ± 2.49 SoC (n = 36) •Age, years: 57.4 ± 14.1 •Male: 27 (75.0%) •Time Sx onset to Tx start, days: 7.38 ± 0.99	RDV + SoC: •Randomized: 41 •Included in analysis: 34 SoC: •Randomized: 41 •Moved to RDV: 1 •Included in analysis: 36
Spinner et al. [34]	Effects of Remdesivir vs Standard Care on Clinical Status at 11 Days in Patients With Moderate COVID-19 A Randomized Clinical Trial	OLRCT	• ≥ 12 years of age •Written informed consent (participants ≥ 18 years of age) •Assent and written informed consent from a parent/legal guardian (participants ≥ 12 and < 18 years of age) •Hospitalized •SARS-CoV-2 infection confirmed by RT-PCR assay ≤ 4 days before randomization •Moderate COVID-19 pneumonia (defined as any radiographic evidence of pulmonary infiltrates and SpO2 > 94% on room air)	•Participation in any other clinical trial of an experimental agent treatment for COVID-19 •Concurrent treatment with other agents with actual or possible direct acting antiviral activity against SARS-CoV-2 < 24 h prior to study •Requiring mechanical ventilation at screening •ALT or AST > 5 times the upper limit of normal •Creatinine clearance < 50 mL/min •Positive pregnancy test •Breastfeeding •Known hypersensitivity to the study drug, the metabolites, or formulation excipient	5-day RDV (n = 191) •D1: 200 mg RDV •D2-D5: 100 mg RDV 10-day RDV (n = 193) •D1: 200 mg RDV •D2-D10: 100 mg RDV Standard care (n = 200)	5-Day RDV (n = 191) •Age, years: 58 (48, 66) •Male: 114 (59.7%) 10-Day RDV (n = 193) •Age, years: 56 (45, 66) •Male: 118 (61.1%) Standard care (n = 200) •Age, years: 57 (45, 66) •Male: 125 (62.5%)	5-day RDV: •Randomized: 199 •Included in analysis: 191 10-day RDV: •Randomized: 197 •Included in analysis: 193 Control: •Randomized: 200 •Included in analysis: 200
Wang et al. [36]	Remdesivir in adults with severe COVID-19: a randomised, double-blind, placebo-controlled, multicentre trial	DBRCT	• ≥ 18 years of age •RT-PCR positive for SARS-CoV-2 •Pneumonia confirmed by chest imaging •SpO2 ≤ 94% on room air or a ratio of arterial oxygen partial pressure to fractional inspired oxygen of ≤ 300 mm Hg •Within 12 days of symptom onset •Eligible patients of child-bearing age (men and women) agreed to take effective contraceptive measures (including hormonal contraception, barrier methods, or abstinence) during the study period and for at least 7 days after the last study drug administration	•Pregnancy or breast feeding •Hepatic cirrhosis •ALT or AST > 5 × ULN •Known severe renal impairment (eGFR < 30 mL/min per 1·73 m^2^) or receipt of continuous renal replacement therapy •Hemodialysis or peritoneal dialysis •Possibility of transfer to a non-study hospital within 72 h •Enrollment into an investigational treatment study for COVID-19 in the 30 days before screening	RDV (n = 158) •D1: 200 mg RDV •D2-D10: 100 mg RDV Control (n = 78) •Equal volume of placebo using same schedule as RDV	RDV (n = 158) •Age, years: 60.0 (57.0, 73.0) •Male: 89 (56%) •WBC, cells/nL 6.2 (4.4, 8.3) •Time Sx onset to Tx start, days: 11 (9, 12) Control (n = 78) •Age, years: 64.0 (53.0, 70.0) •Male: 51 (65%) •WBC, cells/nL 6.4 (4.5, 8.3) Time Sx onset to Tx start, days: 10 (9, 12)	RDV: •Randomized: 158 •Included in analysis: 158 Control: •Randomized: 79 •Included in analysis: 78
Sofosbuvir							
Abbaspour-Kasgari et al. [22]	Evaluation of the efficacy of sofosbuvir plus daclatasvir in combination with ribavirin for hospitalized COVID-19 patients with moderate disease compared with standard care: a single-centre, randomized controlled trial	OLRCT	•18–80 years of age •Positive qualitative RT–PCR for SARS-CoV-2 and/or features consistent with COVID-19 on a chest CT scan was required •Moderate disease on admission were included, which was defined as respiratory rate of < 24/min, arterial O_2_ saturation of > 94% and symptom onset ≤ 8 days prior to admission, together with compatible findings in a chest CT scan •Written informed consent	•Patients with multiorgan failure, active cancer, renal insufficiency (creatine clearance less than 50 mL/min/1.73 m2), anemia (hemoglobin less than 9 g/dL) •Pregnant women or men with a pregnant spouse •Patients treated with amiodarone, phenytoin, phenobarbital, rifabutin or carbamazepine	SOF/DCV + ribavirin (n = 24) •400 mg/60 mg SOF/DCV •1200 mg ribavirin Standard care (n = 24) •400 mg HCQ •400/100 mg LPV/r, 2x/day •1200 mg ribavirin at physician’s discretion	SOF/DCV + ribavirin (n = 24) •Age, years: 45.0 (38.0, 69.0) •Male: 11 (45.8%) •WBC, cells/nL: 6.4 (5.2, 7.7) Standard care (n = 24) •Age, years: 60 (47.5, 68.5) •Male: 7 (29.2%) •WBC, cells/nL: 6.2 (5.9, 9.2)	No patients excluded from analysis
Abbass et al. [46]	Efficacy and safety of sofosbuvir plus daclatasvir or ravidasvir in patients with COVID‐19: A randomized controlled trial	OLRCT	• ≥ 18 years of age •Laboratory‐confirmed symptomatic COVID‐19 determined by PCR assay in any specimen collected < 72 h before randomization •Willing and able to provide written •informed consent •Had the following disease severity grades: moderate (patients with respiratory rate ≥ 20 breaths/min, oxygen saturation •measured through a pulse oximeter [SpO2]˃90% on room air and heart •rate ≥ 90 beats/min), severe (not critical) (patients with clinical signs indicative of severe systematic illness with COVID‐19; such as respiratory •rate ≥ 30/min, heart rate ≥ 125/min, SpO2 ≤ 90% on room air or PaO2/ •FiO2 < 300	•Critically severe COVID‐19 requiring invasive mechanical ventilation at screening •Severe concomitant illness •Hypersensitivity or contraindication to any of the drugs used in the study •Liver cirrhosis or elevated ALT, and/or AST above 3 × ULN •Cardiac ischemia or clinically symptomatic cardiac abnormalities •History of any malignancy •within the last 5 years •History of solid organ or bone •marrow transplantation •Received treatment with any other investigational drug/device or involved in another clinical trial •within 6 months before screening •HIV •Pregnant or breastfeeding	SOF/DCV + SoC (n = 40) •D1-D10: 400 mg/60 mg SOF/DCV •SoC SOF/ravidasvir + SoC (n = 40) •D1-D10: 400 mg/200 mg SOF/ravidasvir •SoC SoC (n = 40)	SOF/DCV + SoC (n = 40) •Age, years: 40.0 ± 6.1 •Male: 22 (55.0%) •SpO_2_ adm: 88.5% ± 5.6 SOF/RDV + SoC (n = 40) •Age, years: 48.0 ± 2.2 •Male: 22 (55.0%) •SpO_2_ adm: 87.8% ± 4.9 SoC (n = 40) •Age, years: 46.0 ± 5.8 •Male: 20 (50.0%) •SpO_2_ adm: 88.7% ± 4.9	No patients excluded from analysis
El-Bendary et al. [49]	Efficacy of combined Sofosbuvir and Daclatasvir in the treatment of COVID-19 patients with pneumonia: a multicenter Egyptian study	OLRCT	• ≥ 18 years of age •Not pregnant •Positive RT-PCR test for •SARS-CoV-2 on nasopharyngeal swab •Evidence of pneumonia on CT chest imaging	•Known allergy •or hypersensitivity to the used medications •Known seizure •disorder •Presence of either active HCV or severe liver disease •(e.g. cirrhosis, elevated liver transaminases > 5 × the upper limit •of the normal range) •Pregnancy or breast-feeding •Cases with history of bone marrow transplant •Glucose 6 phosphate dehydrogenase deficiency •End stage renal disease, psoriasis, porphyria and patients with a known history of long QT syndrome •or current known QTc > 500 ms	SOF/DCV (n = 96) •D1-D14: 400 mg/60 mg SOF/DCV •In combination with the conventional •therapy that included HCQ (400 mg twice daily for 1 day, then 200 mg twice daily for 14 days) Control (n = 78) •Conventional •therapy including HCQ without SOF/DCV	SOF/DCV (n = 96) •Age, years: 52 (37, 67) •Male: 53 (55.2%) •SpO_2_ adm: 89.69% ± 6.54 Control (n = 78) •Age, years: 54 (39, 69) •Male: 42 (53.8%) •SpO_2_ adm: 91.0% ± 5.0	No patients excluded from analysis
Khalili et al. [28]	Efficacy and safety of sofosbuvir/ ledipasvir in treatment of patients with COVID-19; A randomized clinical trial	OLRCT	• ≥ 18 years of age •Admitted to the hospital •Highly suspected (clinical signs/symptoms & imaging findings) or confirmed (positive PCR pharyngeal or nasopharyngeal samples) COVID-19	•History of drug allergy •Decompensated cirrhosis •Severe COVID-19 •Hemodialysis •Pregnant or lactating	SOF/LDP (n = 42) •D1-D10: 400 mg/100 mg SOF/LDP •SoC Control (n = 40) •D1-D10: SoC alone SoC included •D1: HCQ 400 mg 2x/day •D2-D7: HCQ: 200 mg 2x/day •D1-D7: 300 mg/100 mg atazanavir/ritonavir	SOF/LDP (n = 42) •Age, years: 61.5 (46.5, 74.25) •SpO2 adm: 90% (88, 93) •WBC, cells/nL: 6.2 (4.7, 8.4) •Time Sx onset to Tx start, days: 7 (3.75, 10) Control (n = 40) SOF/LDP (n = 42) •Age, years: 63 (53.25, 70.75) •SpO2 adm: 90 (88–93.75) •WBC, cells/nL 5.5 (4.8, 7.5) •Time Sx onset to Tx start, days: 7 (4, 10)	SOF/LDP: •Randomized: 45 •Included in analysis: 42 Standard care: •Randomized: 45 •Included in analysis: 40
Roozbeh et al. [32]	Sofosbuvir and daclatasvir for the treatment of COVID-19 outpatients: a double-blind, randomized controlled trial	DBRCT	• ≥ 18 years of age •Confirmed CT scan findings for COVID-19 •COVID-19 clinical symptoms including fever, cough and fatigue, and positive CRP test •Written informed consent	•SpO_2_ ≤ 93% •Pregnancy •Amiodarone use •Renal failure •Cardiovascular diseases	SOF/DCV + standard care (n = 27) •D1-D7: 400 mg/60 mg SOF/DCV 2x/day Standard care (n = 28) •D1-D7: 200 mg HCQ 2x/day •D1-D6: 500 mg azithromycin •D1-D7: 500 mg naproxen, 2x/day •40 mg pantoprazole tablets	SOF/DCV + standard care (n = 27) •Age, years: 43 (37, 52) •Male: 12 (44.4%) •SpO_2_ (adm): 98% (97, 98) Standard care (n = 28) •Age, years: 47.5 (37, 53) •Male: 14 (50.0%) SpO_2_: 98% (97, 99)	No patients excluded from analysis
Sadeghi et al. [33]	Sofosbuvir and daclatasvir compared with SoC in the treatment of patients admitted to hospital with moderate or severe coronavirus infection (COVID-19): a randomized controlled trial	OLRCT	• ≥ 18 years of age •Positive RT–PCR nasopharyngeal swab and chest CT scan compatible with moderate or severe COVID-19 infection •Signs of severity of disease defined as fever (oral temperature ≥ 37.8 •°C at any one time prior to enrolment) and at least one of respiratory rate > 24/min, SpO_2_ < 94%, or PaO_2_/FiO_2_ratio < 300 mgHg •Onset of symptoms ≤ 8 days •Written informed consent	•Known allergic reaction to the intervention drugs •Pregnant or breastfeeding •Any prior experimental treatment for COVID-19 •HR < 60 bpm •Taking amiodarone •Evidence of multiorgan failure •Requiring invasive mechanical ventilation at screening •eGFR < 50 mL/1.73 m^2^/min	SOF/ DCV + standard care (n = 33) •D1-D14: 400 mg/60 mg SOF/DCV •Standard care Standard care (n = 33) •D1-D14: 200 mg HCQ 2x/day •With or without 200 mg/50 mg LPV/r 2x/day	SOF/DCV + standard care (n = 33) •Age, years: 58 (38–65) •Male: 20 (61%) •SpO_2_ (adm): 91 (89, 92) •WBC, cells/nL: 6.9 (5.6–12.3) Standard care (n = 33) •Age, years: 62 (49, 70) •Male: 14 (42%) •SpO_2_ (adm): 90 (88, 92) •WBC, cells/nL: 10 (6, 12)	No patients excluded from analysis
Sayad et al. [43]	Efficacy and safety of sofosbuvir/velpatasvir versus the standard of care in adults hospitalized with COVID-19: a single-centre, randomized controlled trial	OLRCT	• ≥ 18 years of age •Positive RT-PCR test for SARS-CoV-2 on a nasopharyngeal swab and/or a compatible chest CT scan •SpO_2_ ≤ 93% on ambient air and/or an absolute lymphocyte count of < 1.1 cells/nL	•Pregnancy and breastfeeding •Physician’s decision against enrollment •Conditions that did not allow complete implementation of the protocol •Allergy or hypersensitivity to the drugs used •Severe liver disease (cirrhosis or ALT or AST level > 5 × the upper limit of the normal range) •Use of medications that are contraindicated with the drugs used in this trial •Known HIV infection •Known HCV infection	•SOF/VEL (n = 40) •D1-D10: 400 mg/100 mg SOF/VEL •National SoC •Control (n = 40) •National SoC •National SoC included: •D1: 400 mg HCQ •D1-D10: 400 mg/100 mg LPV/r 2x/day •As needed: supplemental oxygen, non-invasive and invasive ventilation, antimicrobials, vasopressors and corticosteroids	•SOF/VEL (n = 40) •Age, years: 53.6 ± 16.3 •Male: 20 (50%) •WBC, cells/nL 5.7 (4.1, 8.6) •Control: (n = 40) •Age, years: 54.6 ± 19.4 •Male: 24 (60.0%) •WBC, cells/nL 7.5 (6.5, 12)	•SOF/VEL: •Randomized: 40 •Died before treatment: 1 •Control: •Randomized: 40 •Moved to SOF/VEL: 3
**Enisamium**							
Holubovska1 et al. [50]	Enisamium is an inhibitor of the SARS-CoV-2 RNA polymerase and shows improvement of recovery in COVID-19 patients in an interim analysis of a clinical trial	DBRCT	• ≥ 18 years of age •Hospitalized patients with moderate severity of COVID-19 infection diagnosed based on a body temperature of ≥ 37.8 °C and laboratory confirmed presence of SARS-CoV-2 RNA by RT-PCR in pharyngeal swabs or sputum •Informed consent prior to study participation	None reported	Total patients: 373, randomized 1:1 Enisamium (n = ~ 186) •D1-D7: 500 mg enisamium iodide 4x/day Placebo (n = ~ 186) ••D1-D7: 500 mg matching placebo on same schedule	Not reported	No patients excluded from analysis

Data are presented as mean ± standard deviation, median (IQR), or n (%) unless otherwise stated

^*^ statistically different from comparator

AIDS = autoimmune deficiency syndrome; ALT = alanine aminotransferase; ARB = umifenovir (Arbidol); AST = aspartate aminotransferase; B/M = baloxavir/marboxil; BMI = body mass index; CKD = chronic kidney disease; CQ; chloroquine; CT = computed tomography; D# = day #; DB = double-blind; D/C = darunavir/cobicistat; DCV = daclatasvir; ECG = electrocardiogram; ECMO = extracorporeal membrane oxygenation; eGFR = estimated glomerular filtration rate; FVP = favipiravir; HAART = highly active antiretroviral therapy; HCQ = hydroxychloroquine; HCV = hepatitis C virus; HIV = human immunodeficiency virus; IFN = interferon; IU = international units; LDP = ledipasvir; LPV/r = lopinavir/ritonavir; OL = open-label; PaO2/FiO2 = arterial partial pressure of oxygen/fraction of inspired oxygen ratio; QTc = corrected QT interval; RDV = Remdesivir; RCT = randomized controlled trial; RT-PCR = reverse transcriptase polymerase chain reaction; SaO2 = arterial oxygen saturation; SoC = standard of care; SOF = sofosbuvir; SpO2 = oxygen saturation; Sx = symptom; Tx = treatment; ULN = upper limit of normal; VEL = velpatasvir; WBC = white blood cells

**Table 3:** Patient Outcomes

Author	Study name	Primary endpoint	Primary outcomes	Other outcomes	Limitations	Interpretation
Bosaeed et al. [40]	Favipiravir and Hydroxychloroquine Combination Therapy in Patients with Moderate to Severe COVID- 19 (FACCT Trial): An Open-Label, Multicenter, Randomized, Controlled Trial	•Time to clinical •improvement •Defined as the time from randomization to an improvement of two points on a seven-category ordinal scale or live discharge from the hospital, whichever came first	HCQ + FVP (n = 125) •Time to clinical improvement, days: 9 (8, 12) SoC (n = 129) •Time to clinical improvement, days: 7 (6, 10)	HCQ + FVP (n = 125) •Negative SARS-CoV-2 on (RT-PCR) by day 28: 25 (32.1%) •Requirement of ICU admission: 33 (26.4%) •Requirement of MV: 21 (16.8%) •Duration of hospital stay, days: 9 (95% CI: 8, 12) •28-day mortality: 9 (7.6%) SoC (n = 129) •Negative SARS-CoV-2 on (RT-PCR) by day 28: 23 (29.5%) •Requirement of ICU admission: 26 (20.2%) •Requirement of MV: 20 (15.5%) •Duration of hospital stay, days: 8 (95% CI: 7, 10) •28-day mortality: 13 (10.3%)	•Open-label design without a placebo group •Only included •hospitalized patients •High number of follow-up SARS-CoV-2 (RT- •PCR) tests were not obtained because of the limited resources and variable practices •Premature termination could also have led to an •increased data censoring related to the clinical •outcome •SoC group included patients treated with other antivirals	HCQ and FVP combination therapy plus SoC did not achieve a higher efficacy than SoC alone in patients hospitalized with moderate-to-severe COVID-19. [9 (8, 12) vs. 7 (6, 10) p = 0.29]
Chen et al. [42]	Favipiravir versus Arbidol for COVID-19: A Randomized Clinical Trial	•Clinical recovery rate at 7 days from the beginning of treatment •Clinical recovery was defined as continuous (> 72 h) recovery	FVP (n = 116) •Clinical recovery rate oD7: 71 (61.21%) ARB (n = 120) •Clinical recovery rate oD7: 62 (51.67%)	FVP (n = 116) •Incidence of AOT or NMV: 21 (18.1%) •Respiratory failure: 1 (0.9%) ARB (n = 120) •Incidence of AOT or NMV: 27 (22.5%) •Respiratory failure: 4 (3.3%)	•No clinically proven effective antiviral drug or placebo as the control arm •Observation time frame was limited •Did not require positive nucleic acid test in inclusion criteria	FVP did not improve clinical recovery but exhibited better symptom relief than ARB. [71 (61.21) vs. 62 (51.67) p = 0.1396]
Dabbous et al. [42]	Efficacy of favipiravir in COVID-19 treatment: a multi-center randomized study	•Mortality rate •Need for MV	FVP (n = 44) •Mortality: 1 (2.3%) •Need for MV: 0 (0.0%) CQ (n = 48) •Mortality: 2 (4.2%) •Need for MV: 4 (8.3%)	FVP (n = 44) •Duration of hospital stay, days: 13.29 ± 5.86 •SpO_2_: o100-95%: 40 (90.9%) o95-90%: 4 (9.1%) o < 90%: 0 (0) CQ (n = 48) •Duration of hospital stay, days: 15.89 ± 4.75 •SpO_2_: o100-95%: 37 (77.1%) o95-90%: 9 (18.8%) o < 90%: 2 (4.2%)	•Not blinded •No standard care control •Did not examine need for ICU admission, mortality or the viremic response •Included only COVID-19 patients who were mildly or moderately ill and therefore had a better prognosis than severely or critically ill patients	FVP is a promising drug for treatment of COVID-19 that might decrease the hospital stay and the need for MV Mortality rate: [1 (2.3) vs. 2 (4.2) p = 1.00]
Doi et al. [48]	A Prospective, Randomized, Open-Label Trial of Early versus Late Favipiravir Therapy in Hospitalized Patients with COVID-19	•Viral clearance by day 6	Early treatment FVP (n = 36) •SARS-CoV-2 clearance by day 6: 66.7% Late treatment FVP (n = 33) •SARS-CoV-2 clearance by day 6: 56.1%	Early treatment FVP (n = 36) •SARS-CoV-2 clearance by day 10: 86.1% •50% logarithmic reduction in the SARS-CoV-2 viral load by day 6: 94.4% •Median time until SARS-CoV-2 clearance by local RT-PCR: 12.8 •Disease progression or death (n = 44): 0.0 Late treatment FVP (n = 33) •SARS-CoV-2 clearance by day 10: 83.1% •50% logarithmic reduction in the SARS-CoV-2 viral load by day 6: 78.8% •Median time until SARS-CoV-2 clearance by local RT-PCR: 17.8 •Disease progression or death (n = 44): 0.0	•Small sample size •Unexpected high frequency of a negative RT-PCR at the time of enrollment likely underpowered the study •Open-label study design •Staggered treatment design where all patients eventually received FVP, adopted due to the unavailability of placebo at the time of study conception, made it difficult to interpret outcome differences beyond the sixth day •Only recruited asymptomatic to mildly symptomatic COVID-19 patients •Not known whether early treatment had any impact on replication-competent viruses	Administration of FVP did not significantly improve viral clearance in the first 6 days, but there was a trend toward earlier viral clearance with the agent. FVP was associated with numerical reduction in time to defervescence, and a significant improvement in fever was observed the day after starting therapy, compared with findings with no therapy. [66.7 (95% CI, 51.4 to 81.2) vs. 56.1 (95% CI, 0.764 to 2.623) HR = 1.416 (0.764–2.623)]
Lou et al. [30]	Clinical Outcomes and Plasma Concentrations of Baloxavir Marboxil and Favipiravir in COVID-19 Patients: An Exploratory Randomized, Controlled Trial	•Viral negative rate at 14 days •Viral negative was defined as two consecutive RT-PCR tests with undetectable viral RNA •Time from randomization to clinical improvement •Improvement was defined as either increase by two points on NEWS2 or discharge from the hospital	Total (n = 29) •Viral negative, n (%) oD7: 15 (51.7%) oD14: 24 (82.8%) B/M (n = 10) •Viral negative, n (%) oD7: 6 (60.0%) oD14: 7 (70.0%) FVP (n = 9) •Viral negative, n (%) oD7: 4 (44.4%) oD14: 7 (77.8%) Control (n = 10) •Viral negative, n (%) oD7: 5 (50.0%) oD14: 10 (100.0%)	Total (n = 29) •Incidence of MV: 1 (3%) B/M (n = 10) •Incidence of MV: 0 FVP (n = 9) •Incidence of MV: 0 Control (n = 10) •Incidence of MV: 1 (10)	•Small sample size •Subjects were all under treatment with other medication •The poor correlation could be due to the delay between infection and treatment initiation •Patients in FVP group showed oldest average age and shortest time from symptom onset to randomization, even though, the clinical performance of FVP group was not inferior to the other two groups •Not blinded	No extra benefit to COVID-19 treatment was observed when adding B/M or FVP to standard care Viral negative rate at 14 days: [7 (70) vs. 7 (77) vs. 10 (100)] Time from randomization to clinical improvement: [14 (6–49) vs. 14 (6–38) vs. 15 (6–24)]
Shinkai et al. [52]	Efficacy and Safety of Favipiravir in Moderate COVID- 19 Pneumonia Patients without Oxygen Therapy: A Randomized, Phase III Clinical Trial	•Composite outcome defined as the time to •improvement in temperature, SpO_2_, and findings on chest imaging, and recovery to SARS-CoV-2-negative	FVP (n = 107) •Number of patients who improved: 81 •Median time to improvement: 11.9 Placebo (n = 49) •Number of patients who improved: 28 •Median time to improvement: 14.7	FVP (n = 107) •Number of patients who improved: •Temperature: 70 •SpO_2_: 48 •Chest imaging: 95 •Median time to improvement: •Temperature: 2.0 •SpO_2_: 2.9 •Chest imaging: 4.8 •Number of patients with undetectable SARS-CoV-2: 87 •Median time to recovery, SARS-CoV-2: 11.0 Placebo (n = 49) •Number of patients who improved: •Temperature: 30 •SpO_2_: 26 •Chest imaging: 35 •Median time to improvement: •Temperature: 2.1 •SpO_2_: 2.7 •Chest imaging: 5.7 •Number of patients with undetectable SARS-CoV-2: 31 •Median time to recovery, SARS-CoV-2: 12.1	•Single-blind design •Virological •investigations were measured solely by •nasopharyngeal swabs, despite targeting COVID-19 patients with pneumonia •Difficulty in recruiting only suitable patients of early-onset for evaluating antiviral drug efficacy •Only COVID-19 patients with moderate pneumonia •(SpO_2_ ≥ 94%) •Primary endpoint based on COVID-19 patient discharge criterion at that time and cannot be directly •applied to the current criterion	FVP may be one of options for moderate COVID-19 pneumonia treatment. However, the risk of adverse events, including hyperuricemia, should be carefully considered. (11.9 vs. 14.7 p = 0.0136)
Solaymani-Dodaran et al. [44]	Safety and efficacy of Favipiravir in moderate to severe SARS-CoV-2 pneumonia	•Number of admissions to the intensive •care unit	FVP (n = 190) •ICU admission: 31 (16.3%) LPV/r (n = 183) •ICU admission: 25 (13.7%)	FVP (n = 190) •In-hospital mortality: 26 (13.7%) •Intubation: 27 (14.2%) •Length of hospital stay, days (n = 153): 7 (4, 9) •Survival time till clinical recovery, days (n = 185): 6 (4, 10) LPV/r (n = 183) •In-hospital mortality: 21 (11.5%) •Intubation: 17 (9.3%) •Length of hospital stay, days (n = 150): 6 (4, 10) •Survival time till clinical recovery, days (n = 182): 6 (4, 10)	•Not blinded •No control group without antivirals	No clinical benefit from a treatment regimen based on FVP in moderate to severe cases of SARS-CoV-2 over a treatment regimen based on LPV/r. [31 (16.3) vs. 25 (13.7) p = 0.47]
Udwadia et al. [35]	Efficacy and safety of favipiravir, an oral RNA-dependent RNA polymerase inhibitor, in mild-to-moderate COVID-19: A randomized, comparative, open-label, multicenter, phase 3 clinical trial	•Time from randomization to the cessation of oral shedding of the SARS-Cov-2 virus •28 days maximum •Defined as a negative RT-PCR result for both oropharyngeal and nasopharyngeal swabs	FVP (n = 72) •Time to cessation of SARS-CoV-2 oral shedding: oNumber of events: 70 (97.2%) oTime to event, median days: 5.0 Control (n = 75) •Time to cessation of SARS-CoV-2 oral shedding: oNumber of events: 68 (90.7%) oTime to event, median days: 7.0	FVP (n = 72) •Time to clinical cure: oNumber of events: 51/53 (96.2%) oTime to event, median days: 3.0 •Time to hospital discharge: oNumber of events: 70/72 (97.2%) oTime to event, median days: 9.0 Control (n = 75) •Time to clinical cure: oNumber of events: 46/49 (93.9%) oTime to event, median days: 5.0 •Time to hospital discharge: oNumber of events: 68/75 (90.7%) oTime to event, median days: 10.0	•Primary endpoint was confounded by interpretation issues with RT-PCR positivity and its lack of correlation with clinical cure •Impact of RT-PCR assay variables such as cycle time was not evaluated •Hazard ratios observed much smaller than previously reported •Open-label design	Despite failure to achieve statistical significance on the primary endpoint of time to RT-PCR negativity, early administration of oral FVP may reduce the duration of clinical signs and symptoms in patients with mild-to-moderate COVID-19, as demonstrated by the significantly decreased time to clinical cure. [5 (95% CI: 4–7) vs. 7 (95% CI 5–8) p = 0.129]
Zhao et al. [45]	Favipiravir in the treatment of patients with SARS-CoV-2 RNA recurrent positive after discharge: A multicenter, open-label, randomized trial	•Time to achieve consecutive twice (intervals of more than 24 h) negative RT-PCR result for SARS-CoV-2 RNA in nasopharyngeal swab and sputum sample	FVP (n = 36) •SPD (SARS-CoV-2 RNA positive duration) (days): 28.3 ± 16.6 •Proportion of RNA PCR turning negative: 80.6% (29/36) Control (n = 19) •SPD (SARS-CoV-2 RNA positive duration) (days): 27.8 ± 11.3 •Proportion of RNA PCR turning negative: 52.6% (10/19)	FVP (n = 36) •Mortality: 0 (0) •CRP change from baseline: 4.0 ± 9.1 mg/L to 1.5 ± 2.1 mg/L •CD3 + Lymphocyte (count/μL): •D0: 1192.8 ± 444.6 •D15: 1074.4 ± 229.6 •D30: 1094.3 ± 298.9 •CD4 + Lymphocyte (count/μL): •D0: 719.1 ± 226.6 •D15: 484.1 ± 177.4 •D30: 571.8 ± 108.9 •CD8 + Lymphocyte (count/μL): •D0: 473.7 ± 218.5 •D15: 361.9 ± 192.2 •D30: 538 ± 213.7 Control (n = 19) •Mortality: 0 (0) •CRP change from baseline: 2.0 ± 2.8 mg/L to 1.8 ± 2.7 mg/L •CD3 + Lymphocyte (count/μL): •D0: 1159.2 ± 280.7 •D15: 1046.6 ± 275.5 •D30: 778 ± 173.5 •CD4 + Lymphocyte (count/μL): •D0: 672.5 ± 120.2 •D15: 624.7 ± 185.7 •D30: 505.8 ± 151.4 •CD8 + Lymphocyte (count/μL): •D0: 402.2 ± 168.8 •D15: 323.1 ± 93.1 •D30: 334.5 ± 115.6	•Small sample size •Trial was not blinded •Followed up all the patients for only 30 days, and it is not clear whether these patients will return to positive again •Not been able to obtain the Ct value of the dynamic changes of SARS-CoV-2 RNA in patients •Presence of few symptomatic patients in this study, and only mild symptoms, prevents from demonstrating a clear clinical benefit of FVP •Hospital admission is mandatory in PCR positive patients in China, and discharge is not allowed meanwhile PCR is still positive, but these measures are not followed worldwide, so the benefits of treatment may not be widespread in other settings	FVP was safe and superior to control in shortening the duration of viral shedding in SARS-CoV-2 RNA recurrent positive after discharge. [27.8 vs. 28.3 HR = 2.1 (95% CI 1.1–4.0) p = 0.038]
Ader et al. [37]	An open-label randomized, controlled trial of the effect of lopinavir/ritonavir, lopinavir/ ritonavir plus IFN-β-1a and hydroxychloroquine in hospitalized patients with COVID-19	•Clinical status at day 15, measured by the WHO 7-point ordinal scale •7-point ordinal scale: o1. Not hospitalized/no olimitations on activities o2. Not hospitalized, limitation oon activities o3. Hospitalized, not requiring osupplemental oxygen o4. Hospitalized, requiring osupplemental oxygen o5. Hospitalized, on non-invasive ventilation or high flow oxygen device o6. Hospitalized, on IMV or ECMO o7. Death	LPV/r + standard of care(n = 145), moderate (n = 94)/severe (n = 51): •1: 21 (22.3%)/ 1 (2.0%) •2: 36 (38.3%)/ 2 (3.9%) •3: 16 (17.0%)/ 5 (9.8%) •4: 9 (9.6%)/ 9 (17.6%) •5: 2 (2.1%)/ 1 (2.0%) •6: 7 (7.4%)/ 29 (56.9%) •7: 3 (3.2%)/ 4 (7.8%) LPV/r + IFN + standard of care (n = 145), moderate (n = 91)/severe (n = 54): •1: 20 (22.0%)/ 0 (0.0%) •2: 35 (38.5%)/ 1 (1.9%) •3: 13 (14.3%)/ 5 (9.3%) •4: 9 (9.9%)/ 6 (11.1%) •5: 2 (2.2%)/ 4 (7.4%) •6: 9 (9.9%)/ 28 (51.9%) •7: 3 (3.3%)/ 10 (18.5%) HCQ + standard of care (n = 145), moderate (n = 93)/severe (n = 52): •1: 20 (21.5%)/ 1 (1.9%) •2: 34 (36.6%)/ 7 (13.5%) •3: 18 (19.4%)/ 7 (13.5%) •4: 11 (11.8%)/ 6 (11.5%) •5: 1 (1.1%)/ 3 (5.8%) •6: 5 (5.4%)/ 25 (48.1%) •7: 4 (4.3%)/ 3 (5.8%) Control (n = 148), moderate (n = 94)/severe (n = 54): •1: 23 (24.5%)/ 1 (1.9%) •2: 41 (43.6%)/ 6 (11.1%) •3: 7 (7.4%)/ 5 (9.3%) •4: 12 (12.8%)/ 10 (18.5%) •5: 1 (1.1%)/ 2 (3.7%) •6: 6 (6.4%)/ 24 (44.4%) •7: 4 (4.3%)/ 6 (11.1%)	LPV/r + standard of care(n = 145), moderate (n = 94)/severe (n = 51): •Death within 28 days: 4 (4.3%)/ 10 (19.6%) LPV/r + IFN + standard of care (n = 145), moderate (n = 91)/severe (n = 54): •Death within 28 days: 4 (4.4%)/ 13 (24.1%) HCQ + standard of care (n = 145), moderate (n = 93)/severe (n = 52): •Death within 28 days: 6 (6.5%)/ 5 (9.6%) Control (n = 148), moderate (n = 94)/severe (n = 54): •Death within 28 days: 5 (5.3%)/ 7 (13.0%)	•Open-labelled design •Did not target patients at the early phase of the disease •Did not include arms testing anti-inflammatory agents that could be used as part of the standard of care arm •Standard of care •underwent substantial changes over time	In patients admitted to hospital with COVID-19, LVP/r, LVP/r plus IFN-β-1a and HCQ were not associated with clinical improvement at day 15 and day 29, nor reduction in viral shedding. [aOR 0.83 (95% CI 0.55–1.26 p = 0.39) vs. aOR 0.69 (95% CI 0.45–1.04 p = 0.08) vs. aOR 0.93 (95% CI 0.62–1.41 p = 0.75)]
Alavi Darazam et al. [47]	Umifenovir in hospitalized moderate to severe COVID-19 patients: A randomized clinical trial	•Time clinical improvement evaluated based on improvement of two points of the seven-category ordinal scale (recommended by the World Health Organization) or discharge from the hospital, •whichever came first	LPV/r + HCQ + IFN-β-1a + ARB (n = 51) •Time to clinical •improvement: 9 (5–11) Control (n = 50) •Time to clinical •improvement, median: 7 (4–10)	LPV/r + HCQ + IFN-β-1a + ARB (n = 51) •Mortality at D •21: 17 (33.3%) •ICU adm: 51 (100.0%) •IMV: 17 (33.3%) Control (n = 50) •Mortality at D •21: 19 (38.0%) •ICU adm: 50 (100.0%) •IMV: 14 (28.0%)	•Not blinded •38 patients unable to complete treatment course of administration because of liver enzyme elevation •The trial was •conducted on hospitalized patients with moderate-severe COVID-19 and the effectiveness of umifenovir in patients with mild Covid-19 not •evaluated	Additive ARB was not effective in shortening the duration of SARS-CoV-2 in severe patients and improving the prognosis in non-ICU patients. [9 (5–11) vs. 7 (4–10) p = 0.22]
Arabi et al. [38]	Lopinavir-ritonavir and hydroxychloroquine for critically ill patients with COVID-19: REMAP-CAP randomized controlled trial	•Ordinal scale of organ support-free days	LPV/r (n = 225) •Organ support-free days: 4 (− 1, 15) HCQ (n = 50) •Organ support-free days: 0 (− 1, 9) Combination therapy (n = 27) •Organ support-free days: − 1 (− 1, 7) Control (n = 362) •Organ support-free days: 6 (− 1, 16)	LPV/r (n = 225) •90-day survival, adjusted HR: 0.83 (95% CI: 0.65, 1.07) •Respiratory support-free days: 3 (− 1, 15) •Time to hospital discharge, adjusted HR: 0.83 (95% CI: 0.68, 0.99) •Progression to IMV, ECMO or death: 89/176 (50.6%) HCQ (n = 50) •90-day survival, adjusted HR: 0.71 (95% CI: 0.45, 0.97) •Respiratory support-free days: 0 (− 1, 9) •Time to hospital discharge, adjusted HR: 0.76 (95% CI: 0.56, 0.97) •Progression to IMV, ECMO or death: 17/24 (70.8%) Combination therapy (n = 27) •90-day survival, adjusted HR: 0.58 (95% CI: 0.36, 0.92) •Respiratory support-free days: −1 (− 1, 7) •Time to hospital discharge, adjusted HR: 0.63 (95% CI: 0.42, 0.89) •Progression to IMV, ECMO or death: 11/14 (78.6%) Control (n = 362) •90-day survival, adjusted HR: 1 •Respiratory support-free days: 5 (− 1, 16) •Time to hospital discharge, adjusted HR: 1 •Progression to IMV, ECMO or death: 107/239 (44.8%)	•Data on the bioavailability of dissolved or crushed •LPV/r tablets in critically ill patients are limited •Open-label design	Among critically ill patients with COVID-19, treatment with LPV/r, HCQ, or combination therapy resulted in worse outcomes compared to no antiviral therapy. [4 (-1, 15) vs. 0 (-1, 9) vs. -1 (-1, 7) vs. 6 (-1, 16)]
Cao et al. [24]	A Trial of Lopinavir–Ritonavir in Adults Hospitalized with Severe Covid-19	•Time to clinical improvement, defined as the time from randomization to either an improvement of two points on a seven-category ordinal scale or discharge from the hospital, whichever came first	LPV/r (n = 99) •Time to clinical improvement, days: 16.0 (13.0, 17.0) Control (n = 100) •Time to clinical improvement, days: 16.0 (15.0, 18.0)	LPV/r (n = 99) •28-day mortality: 19 (19.2%) •Clinical improvement: •D7: 6 (6.1%) •D14: 45 (45.5%) •D28: 78 (78.8%) •Hospital stay (days): 14 (12, 17) •Duration of IMV: 4 (3, 7) Control (n = 100) •28-day mortality: 25 (25.0%) •Clinical improvement: •D7: 2 (2.0%) •D14: 30 (30.0%) •D28: 70 (70.0%) •Hospital stay (days): 16 (13, 18) •Duration of IMV: 5 (3, 9)	•Not blinded •Characteristics of the patients at baseline were generally balanced across the two groups, but the somewhat higher throat viral loads in the LPV/r group raise the possibility that this group had more viral replication •Do not have data on the LPV exposure levels in patients	In hospitalized patients with severe COVID-19, LPV/r showed no benefit compared to standard care. [16 vs. 16 HR = 1.31, 95% CI (0.95–1.85), p = 0.09]
Li et al. [29]	Efficacy and safety of lopinavir/ritonavir or arbidol in adult patients with mild/moderateCOVID-19: an exploratory randomized controlled trial	•Rate of positive-to-negative conversion of SARS-CoV-2 nucleic acid	LPV/r (n = 34) •Positive-to-negative conversion of SARS-CoV-2 nucleic acid by pharyngeal swab •D7: 12 (35.3%) ARB (n = 35) •Positive-to-negative conversion of SARS-CoV-2 nucleic acid by pharyngeal swab •D7: 13 (37.1%) Control (n = 17) •Positive-to-negative conversion of SARS-CoV-2 nucleic acid by pharyngeal swab •D7: 7 (41.2%)	LPV/r (n = 34) •Positive-to-negative conversion of SARS-CoV-2 nucleic acid by pharyngeal swab •D14: 29 (85.3%) •Time of positive-to-negative conversion of SARS-CoV-2 nucleic acid in pharyngeal swab (days): 9.0 ± 5.0 •Conversion rate from moderate to severe/critical clinical status: 8 (23.5%) ARB (n = 35) •Positive-to-negative conversion of SARS-CoV-2 nucleic acid by pharyngeal swab •D14: 32 (91.4%) •Time of positive-to-negative conversion of SARS-CoV-2 nucleic acid in pharyngeal swab, days: 9.1 ± 4.4 •Conversion rate from moderate to severe/critical clinical status: 3 (8.6%) Control (n = 17) •Positive-to-negative conversion of SARS-CoV-2 nucleic acid by pharyngeal swab oD14: 13 (76.5%) •Time of positive-to-negative conversion of SARS-CoV-2 nucleic acid in pharyngeal swab, days: 9.3 ± 5.2 •Conversion rate from moderate to severe/critical clinical status: 2 (11.8%)	•Small sample size •Did not include severely or critically ill patients or patients at increased risk of poor outcomes with many comorbidities •Not completely blinded	LPV/r and ARB therapy show little benefit for improving clinical outcome in hospitalized patients with mild to moderate COVID-19 compared to supportive care. [35.3 vs. 37.1 vs. 41.2 p = 0.966]
Nojomi et al. [31]	Effect of Arbidol (Umifenovir) on COVID-19: a randomized controlled trial	•Duration of hospitalization •Time to clinical improvement	LPV/r (n = 50) •Duration of hospitalization, days: 9.6 ± 5.2 •Time to clinical improvement: 3.1 ± 1.4 ARB (n = 50) •Duration of hospitalization, days: 7.2 ± 4.7 •Time to clinical improvement: 2.7 ± 1.1	LPV/r (n = 50) •30-day mortality: 2 (4.0%) •IMV: 2 (4.0%) ARB (n = 50) •30-day mortality: 1 (2.0%) •IMV: 3 (6.0%)	•Not blinded •Treatments were given in combination with HCQ •Small sample sizes for disease severity subgroups	ARB significantly shortens duration of hospitalization compared to LPV/r in patients with COVID-19 Duration of hospitalization: (7.2 vs. 9.6 p = 0.02) Time to clinical improvement: (2.7 vs. 3.1)
RECOVERY collaborative group [26]	Lopinavir–ritonavir in patients admitted to hospital with COVID-19 (RECOVERY): a randomised, controlled, open-label, platform trial	•28-day all-cause mortality	LPV/r (n = 1616) •28-day mortality: 374 (23%) Standard care (n = 3424) •28-day mortality: 767 (22%)	LPV/r (n = 1616) •Discharged from hospital within 28 days: 1113 (69%) •IMV: 152/1556 (10%) •Death: 350/1556 (22%) Standard care (n = 3424) •Discharged from hospital within 28 days: 2382 (70%) •IMV: 279/3280 (9%) •Death: 712/3280 (22%)	•Not blinded •Did not collect detailed information on non-serious adverse reactions or reasons for stopping treatment •Did not collect information on physiological, laboratory, or virological parameters •Very few intubated patients with COVID-19 were enrolled in this study as there were difficulties in administering treatment to patients who could not swallow	LPV/r was not associated with reduction in 28-day mortality, duration of hospital stay, or risk of progression to IMV or death. [23 vs. 22, 95% CI (0.91–1.17) p = 0.60]
Reis et al. [27]	Effect of Early Treatment With Hydroxychloroquine or Lopinavir and Ritonavir on Risk of Hospitalization Among Patients With COVID-19 The TOGETHER Randomized Clinical Trial	•COVID-19-associated •hospitalization and death 90 days after randomization	HCQ (n = 214) •COVID-19 hospitalization: 8 (3.7%) •Death: 0 (0.0%) LPV/r (n = 244) •COVID-19 hospitalization: 14 (5.7%) •Death: 2 (0.8%) Placebo (n = 227) •COVID-19 hospitalization: 11 (4.8%) •Death: 1 (0.4%)	HCQ (n = 214) •All-cause hospitalization: 11 (5.1%) •Time to viral clearance (n = 185): 97 (52.4%) LPV/r (n = 244) •All-cause hospitalization: 16 (6.6%) •Time to viral clearance (n = 201): 125 (62.2%) Placebo (n = 227) •All-cause hospitalization: 12 (5.3%) •Time to viral clearance (n = 195): 112 (57.4%)	•Found a low rate of hospitalizations, even though the population had risk factors for developing serious COVID-19 and median (range) age of 53 (18–94) years	No clinical benefit to support the use of either HCQ or LPV/r in an outpatient population Hospitalization: [8 (3.7) vs. 14 (5.7) vs. 11 (4.8)] Death: [0 (0) vs. 2 (0.8) vs. 1 (0.4)]
Barratt-Due et al. [39]	Evaluation of the Effects of Remdesivir and Hydroxychloroquine on Viral Clearance in COVID-19: A Randomized Trial	•All-cause, in- •hospital mortality	RDV (n = 42) •Mortality during •hospitalization: 7.1% (95% CI: 1.8 to 17.5) RDV control (n = 57) •Mortality during •hospitalization: 7.0% (95% CI: 2.2 to 15.6) HCQ (n = 52) •Mortality during •hospitalization: 7.5% (95% CI: 2.4 to 16.7) HCQ control (n = 54) •Mortality during •hospitalization: 3.6% (95% CI: 0.6 to 10.6)	RDV (n = 42) •Admission to ICU during •hospitalization: 19.0% (95% CI: 9.2 to 32.6) •MV •during hospitalization: 9.5% (95% CI: 3.1 to 20.8) RDV control (n = 57) •Admission to ICU during •hospitalization: 19.3% (95% CI: 10.5 to 30.8) •MV •during hospitalization: 7.0% (95% CI: 2.2 to 15.6) HCQ (n = 52) •Admission to ICU during •hospitalization: 22.6% (95% CI: 12.8 to 35) •MV •during hospitalization: 15.1% (95% CI: 7.2 to 26.3) HCQ control (n = 54) •Admission to ICU during •hospitalization: 16.1% (95% CI: 8.1 to 27.1) •MV •during hospitalization: 10.7% (95% CI: 4.4 to 20.5)	•Not blinded •Relatively few •patients were included, and CIs were wide enough to include moderate effects •Not all data were available from all patients at all •time points •Most of the patients did not receive the full treatment length •of the tested medication due to hospital discharge	Neither RDV nor HCQ affected viral clearance in hospitalized patients with COVID-19 [7.1 vs. 7.0 vs. 7.5 vs. 3.6]
Beigel et al. [23]	Remdesivir for the Treatment of Covid-19—Final Report	•Time to recovery •Defined by either discharge from the hospital or hospitalization for infection-control purposes only	RDV (n = 541) •Time to recovery: 10 (9, 11) Control (n = 521) •Time to recovery: 15 (13, 18)	RDV (n = 541) •Recovery: 399 (73.8%) •29-day mortality: 59 (10.9%) •Time to clinical improvement, one category on ordinal scale, days 7.0 (6.0, 8.0) •Duration of initial hospitalization, days: 12 (6, 28) •New use of MV or ECMO: 52/402 (12.9%) Control (n = 521) •Recovery: 352 (67.6%) •29-day mortality: 77 (14.8%) •Time to clinical improvement, one category on ordinal scale, days: 9.0 (8.0, 11.0) •Duration of initial hospitalization, days: 17 (8, 28) •New use of MV or ECMO: 82/364 (22.5%)	•Training, site initiation visits, and monitoring visits often were performed remotely due to restricted travel and hospital restriction of entrance of nonessential personnel •Research staff were often assigned other clinical duties and staff illnesses strained research resources •Many sites did not have adequate supplies of personal protective equipment and trial-related supplies, such as swabs	RDV shortens time to recovery in hospitalized COVID-19 patients with evidence of infection in the lower respiratory tract [10 days vs. 15 days, p < 0.001]
Goldman et al. [53]	Remdesivir for 5 or 10 Days in Patients with Severe Covid-19	•Clinical status assessed on D14 on a 7-point ordinal scale o1. death o2. hospitalized, receiving IMV or ECMO o3. hospitalized, receiving noninvasive ventilation or high-flow oxygen devices o4. hospitalized, requiring low-flow supplemental oxygen o5. hospitalized, not requiring supplemental oxygen but receiving ongoing medica care (related or not related to Covid-19); o6. hospitalized, requiring neither supplemental oxygen nor ongoing medical care (other than that specified in the protocol for RDV administration) o7. not hospitalized	5-day RDV (n = 200) •Clinical status at day 14 on the 7-point ordinal scale: •1: 16 (8.0%) •2: 16 (8.0%) •3: 9 (4.5%) •4: 19 (9.5%) •5: 11 (5.5%) •6: 9 (4.5%) •7: 120 (60.0%) 10-day RDV (n = 197) •Clinical status at day 14 on the 7-point ordinal scale: •1: 21 (10.5%) •2: 33 (16.5%) •3: 10 (5.0%) •4: 14 (7.0%) •5: 13 (6.5%) •6: 3 (1.5%) •7: 103 (51.5%)	5-day RDV (n = 200) •Time to clinical improvement (median day of 50% cumulative incidence): 10 •Time to recovery (median day of 50% cumulative incidence): 10 10-day RDV (n = 197) •Time to clinical improvement (median day of 50% cumulative incidence): 11 •Time to recovery (median day of 50% cumulative incidence): 11	•Not blinded •Did not have SARS-CoV-2 viral-load results during and after treatment, owing to the variability in local access to testing and practices across the global sites	No significant difference was found between a 5-day course and a 10-day course of RDV in patients with severe Covid-19 not requiring MV [65.2 vs. 57.1, 95% CI (1.16–1.90) p = 0.002]
Mahajan et al. [51]	Clinical outcomes of using remdesivir in patients with moderate to severe COVID-19: A prospective randomised study	•Improvement in clinical outcomes	RDV + standard of care (n = 34) •Did not require hospitalization: 2 (5.9%) Standard of care (n = 36) •Did not require hospitalization: 3 (8.3%)	RDV + standard of care (n = 34) •Hospitalized, but did not require supplemental oxygen: 0 (0.0%) •Hospitalized, required supplemental oxygen: 4 (11.8%) •Required high-flow oxygen or non-invasive ventilation: 19 (55.9%) •Required or received MV: 4 (11.8%) •Death: 5 (14.7%) Standard of care (n = 36) •Hospitalized, but did not require supplemental oxygen: 0 (0.0%) •Hospitalized, required supplemental oxygen: 6 (16.7%) •Required high-flow oxygen or non-invasive ventilation: 22 (61.1%) •Required or received MV: 2 (5.6%) •Death: 3 (8.3%)	•All study cases were of moderate to severe disease category •Did not grade the adverse events •Did not give placebo injection in the no-RDV group •Not blinded •Small sample size	RDV therapy for five days did not produce improvement in clinical outcomes in moderate to severe COVID-19 cases [2 (5.9) vs. 3 (8.3) p = 0.749]
Spinner et al. [34]	Effect of Remdesivir vs Standard Care on Clinical Status at 11 Days in Patients With Moderate COVID-19 A Randomized Clinical Trial	•Difference in clinical status distribution	10-day RDV (n = 193) •Difference in clinical status distribution vs standard care: p = 0.18 5-day RDV (n = 191) •Difference in clinical status distribution vs standard care: OR 1.65 (95% CI: 1.09, 2.48), p = 0.02	10-day RDV (n = 193) •D11 clinical status oDeath: 2 (1.0%) oNot hospitalized: 125 (64.8%) 5-day RDV (n = 191) •D11 clinical status oDeath: 0 (0.0%) oNot hospitalized: 134 (70.2%) Standard care (n = 200) •D11 clinical status oDeath: 4 (2.0%) oNot hospitalized: 120 (60.0%)	•Original protocol written when clinical understanding of disease was limited, so primary end point changed on first day of study enrollment •Open-label design •Virological outcomes (SARS-CoV-2 viral load) not assessed •Other lab parameters that may have aided in identifying predictors of outcomes not collected	5-day course of RDV improved clinical status of moderate COVID-19 patients, but the magnitude of treatment was of questionable clinical relevance [1.65 (1.09–2.48) vs. 1 p = 0.02]
Wang et al. [36]	Remdesivir in adults with severe COVID-19: a randomised, double-blind, placebo-controlled, multicentre trial	•Time to clinical improvement up to day 28 •Defined as the time from randomization to the point of a decline of two levels on a six-point ordinal scale of clinical status (from 1 = discharged to 6 = death) or discharged alive from hospital, whichever came first	RDV (n = 158) •Time to clinical improvement: 21.0 (13.0, 28.0) Control (n = 78) •Time to clinical improvement: 23.0 (15.0, 28.0)	RDV (n = 158) •Clinical improvement rates oD7: 4 (2.5%) oD14: 42 (26.6%) oD28: 103 (65.2%) •D28 mortality: 22 (13.9%) •Duration of IMV, days: 7.0 (4.0, 16.0) •Duration of hospital stay, days: 25.0 (16.0, 38.0) Control (n = 78) •Clinical improvement rates oD7: 2 (2.6%) oD14: 18 (23.1%) oD28: 45 (57.7%) •D28 mortality: 10 (12.8%) •Duration of IMV, days: 15.5 (6.0, 21.0) •Duration of hospital stay, days: 24.0 (18.0, 36.0)	•Insufficient power to detect assumed differences in clinical outcomes •Initiation of treatment late after symptom onset •Frequent use of corticosteroids patients may have promoted viral replication •No answer to whether longer treatment course and higher dose of RDV would be beneficial in patients with severe COVID-19	No benefits were observed with RDV above and beyond that observed with standard therapies in severe COVID-19 patients [21.0 (13.0, 28.0) vs. 23.0 (15.0, 28.0), 95% CI 1.23 (0.87–1.75)]
Abbaspour-Kasgari et al. [22]	Evaluation of the efficacy of sofosbuvir plus daclatasvir in combination with ribavirin for hospitalized COVID-19 patients with moderate disease compared with standard care: a single-centre, randomized controlled trial	•Length of hospital stay	SOF/DCV + ribavirin (n = 24) •Duration of hospitalization, days: 6 (5, 7) Standard care (n = 24) •Duration of hospitalization, days: 6 (5.5, 7.5)	SOF, DCV, ribavirin (n = 24) •Recovery: 24 (100.0%) •Death: 0 (0.0%) •Time to recovery, days: 6 (5, 7) •ICU admission: 0 (0.0%) •ICU duration, days: N/A •IMV: 0 (0.0%) •IMV duration, days: N/A Standard care (n = 24) •Recovery: 21 (87.5%) •Death: 3 (12.5%) •Time to recovery, days: 6 (6, 8) •ICU admission: 4 (16.7%) •ICU duration, days: 2.5 (1.5, 7) •IMV: 4 (16.7%) •IMV duration, days: 2.5 (1.5, 7)	•Median age was higher in the control arm •More patients with diabetes in the control arm •Number of patients not high enough to identify probable beneficial effects on survival •Excluded elderly subject •Not blinded •Not able to analyze biological markers of improvement	There were signs of improved recovery and death rates in the with SOF/DCV + ribavirin, but the sample size was too small to see conclusive differences [6 (5–7) vs. 6 (5.5–7.5) p = 0.398]
Abbass et al. [46]	Efficacy and safety of sofosbuvir plus daclatasvir or ravidasvir in patients with COVID‐19: A randomized controlled trial	•Sum of the counted symptoms at D7 and D10 compared to D3 •Mean change in SpO_2_ from D1 to D10	SOF/DCV + SoC (n = 40) •D7 change in counts of •clinical symptoms, value (SE) (*p* versus SoC: 0.041): − 0.12647 (0.13953) •D10 change in counts of •clinical symptoms, value (SE) (*p* versus SoC: 0.0399): − 0.031655 (0.174262) SOF/ravidasvir + SoC (n = 40) •D7 change in counts of •clinical symptoms, value (SE) (*p* versus SoC: 0.491): − 0.09579 (0.13895) •D10 change in counts of •clinical symptoms, value (SE) (*p* versus SoC: 0.66969): + 0.071006 (0.166456)	SOF/DCV + SoC (n = 40) •D1 SpO_2_: 88.7 ± 4.2 •D10 SpO_2_: 95.8 ± 2.7 SOF/ravidasvir + SoC (n = 40) •D1 SpO_2_: 87.5 ± 6.25 •D10 SpO_2_: 94.52 ± 4.58 SoC (n = 40) •D1 SpO_2_: 87.9 ± 5.8 •D10 SpO_2_: 93.4 ± 3.7	•Small sample size •Open‐label design •Lack of a placebo group	SOF/DCV + SoC was found to improve clinical symptoms, oxygen saturation, and decrease ICU admission. SOF/ravidasvir had no effect relative to SoC alone
El-Bendary et al. [49]	Efficacy of combined Sofosbuvir and Daclatasvir in the treatment of COVID-19 patients with pneumonia: a multicenter Egyptian study	•Rate of clinical/ virological cure	SOF/DCV (n = 96) •Negative PCR D7: 12/24 (50.0%) •Negative PCR D14: 81/96 (84.4%) Control (n = 78) •Negative PCR D7: 9/25 (36.0%) •Negative PCR D14: 37/78 (47.4%)	SOF/DCV (n = 96) •Adm to hospital: 79 (82.3%) •ICU adm: 19 (19.8%) •Duration inside hospital, median (IQR): 8 (9%) •Follow up of WHO assessment scale, improved: 76 (79.2%) Control (n = 78) •Adm to hospital: 49 (62.8%) •ICU adm: 24 (30.8%) •Duration inside hospital, median (IQR): 10 (12%) •Follow up of WHO assessment scale, improved: 57 (73.1%)	•Not blinded	SOF/DCV was effective as a treatment for COVID-19 and was associated with reduced hospital stay, a larger proportion of virological clearance at Day 14 and a trend toward lower mortality [84.4 vs. 47.4 p < 0.01]
Khalili et al. [28]	Efficacy and safety of sofosbuvir/ ledipasvir in treatment of patients with COVID-19; A randomized clinical trial	•Clinical response •Time to clinical •response •Clinical response •was defined as one order decline in disease category •in the five category ordinal scale	SOF/LDP (n = 42) •Clinical response: 38 (90.5%) •Time to clinical response, days: 2 (1, 3.75) Control (n = 40) •Clinical response: 37 (92.5%) •Time to clinical response, days: 4 (2, 5)	SOF/LDP (n = 42) •Duration of hospital stay, days: 4 (2, 9.5) •Duration of ICU stay, days: 6 (4, 11) •14-day mortality: 3 (8.8%) Control (n = 40) •Duration of hospital stay, days: 5 (3.25, 7) •Duration of ICU stay, days: 9 (6, 12) •14-day mortality: 3 (7.5%)	•Not blinded •Follow-up RT-PCR and chest imaging were not possible •Small sample size	SOF/LDP accelerated time to the clinical response, but did not have a significant effect on duration of hospital stay or mortality Clinical Response: [38 (90.48) vs. 37 (92.5) p = 0.65] Time to clinical response (days): [2 (1–3.75) vs. 4 (2.5) p = 0.02]
Roozbeh et al. [32]	Sofosbuvir and daclatasvir for the treatment of COVID-19 outpatients: a double-blind, randomized controlled trial	•Symptom alleviation after 7 days of follow-up	SOF/DCV + standard care (n = 27) •Any symptoms: •D1: 27 (100.0%) •D3: 16 (59.3%) •D5: 12 (44.4%) •D7: 7 (25.9%) Standard care (n = 28) •Any symptoms: •D1: 26 (92.9%) •D3: 15 (53.6%) •D5: 12 (42.9%) •D7: 7 (25.0%)	SOF/DCV + standard care (n = 27) •Hospital admission: 1 (3.7%) •Fatigue D30: 2 (7.4%) •Anosmia D30: 0 (0.0%) •Dyspnea D30: 4 (14.8%) Standard care (n = 28) •Hospital admission: 4 (14.3%) •Fatigue D30: 16/26 (61.5%) •Anosmia D30: 3/26 (11.5%) •Dyspnea D30: 11/26 (42.3%)	•Assessment of symptom outcomes not carried out using an objective grading system •Small sample size	SOF/DCV did not significantly reduce symptoms at 7 days compared to control. However, the intervention significantly reduced the number of patients with fatigue and dyspnea at 1 month [7 (26) vs. 7 (28) p = 1.00]
Sadeghi et al. [33]	Sofosbuvir and daclatasvir compared with standard of care in the treatment of patients admitted to hospital with moderate or severe coronavirus infection (COVID-19): a randomized controlled trial	•Clinical recovery within 14 days of treatment	SOF/DCV + standard care (n = 33) •Clinical recovery ≤ 14 days: 29 (87.9%) Standard care (n = 33) •Clinical recovery ≤ 14 days: 22 (66.7%)	SOF/DCV + standard care (n = 33) •Duration of hospitalization, days: 6 (4, 8) •Time to clinical recovery, days: 6 (4, 10) •IMV: 3 (9.1%) •Death: 3 (9.1%) Standard care (n = 33) •Duration of hospitalization, days): 8 (5, 13) •Time to clinical recovery, days: 11 (6, 17) •IMV: 7 (21.2%) •Death: 5 (15.2%)	•Not blinded •Fewer patients in the treatment arm received LVP/r •Small sample size	SOF/DCV significantly reduced the duration of hospital stay [29 (88) vs. 22 (67) p = 0.076]
Sayad et al. [43]	Efficacy and safety of sofosbuvir/velpatasvir versus the standard of care in adults hospitalized with COVID-19: a single-centre, randomized controlled trial	•28-day mortality	SOF/VEL (n = 40) •All-cause mortality: 3 (7.5%) Control (n = 40) •All-cause mortality: 3 (7.5%)	SOF/VEL (n = 40) •Time to clinical improvement, days: 6 (4, 8) •Duration of hospital stay, days: 6 (5, 8.5) •Time from randomization to death, days: 6 (2, 9) •Need for MV: 1 (2.4%) •Duration of MV—days: 3 (3, 3) •RT-PCR conversion (positive to negative): 6 (15.0%) Control (N = 40) •Time to clinical improvement, days: 7 (4–11) •Duration of hospital stay, days: 7 (5–13) •Time from randomization to death, days: 7 (7, 30) •Need for MV: 3 (8.1%) •Duration of MV, days: 1 (1, 1) •RT-PCR conversion (positive to negative): 4 (10.0%)	•Did not assess viral load •Small sample size •Open-label design	SOF/VEL + SoC did not improve the clinical status or reduce mortality in patients with moderate to severe COVID-19 [3 (7.5) vs. 3 (7.5) p = 1.00]
Holubovska et al. [50]	Enisamium is an inhibitor of the SARS-CoV-2 RNA polymerase and shows improvement of recovery in COVID-19 patients in an interim analysis of a clinical trial	•Time-to-recovery •Defined as improvement in the Severity Rating (SR) baseline status by 2 SR score values (e.g., a change from SR 4 to SR 6)	Enisamium (n = ~ 186) •Mean time-to- •recovery, days: 11.1 Placebo (n = ~ 186) •Mean time-to- •recovery, days: 13.9 days	Enisamium (n = ~ 186) •Maximum time-to-recovery, days: 21 Placebo (n = ~ 186) •Maximum time-to-recovery, days: not reported	•Patient baseline characteristics not reported •Group sizes not directly reported	Enisamium treatment shortens the time to recovery for COVID-19 patients needing oxygen [13.9 vs. 11.1 p = 0.0259]

Data are presented as mean ± standard deviation or median (IQR) unless otherwise stated

*Statistically different from comparator

*Adm* = admission; *ALT =* alanine aminotransferase; *AOT* = ambulatory oxygen therapy; ARB = umifenovir (Arbidol); *AST* = aspartate aminotransferas;, *B/M* = baloxavir/marboxil; *CQ =* chloroquine; *CT* = computed tomography; *D#* = day #; *DB* = double-blind; *DCV =* daclatasvir; *ECMO* = extracorporeal membrane oxygenation; *FVP* = favipiravir; *GI* = gastrointestinal; *HCQ =* hydroxychloroquine; HR = hazard ratio; *ICU* = intensive care unit; *IFN =* interferon; *IMV =* invasive mechanical ventilation; *LDP =* ledipasvir; *LPV/r =* lopinavir/ritonavir; *MV =* mechanical ventilation; *NMV =* non-invasive mechanical ventilation; *OL* = open-label; *OR* = odds ratio; PaO2/FiO2 = arterial partial pressure of oxygen/fraction of inspired oxygen ratio; *QTc* = corrected QT interval; *RDV* = Remdesivir; *RCT =* randomized controlled trial; *RT-PCR =* reverse transcriptase polymerase chain reaction; *rxn =* reaction; *SoC =* standard of care; SOF = sofosbuvir; *SpO2* = oxygen saturation; *VEL* = velpatasvir

### Favipiravir

Favipiravir is an antiviral used to treat influenza in Japan. It is a purine analog that inhibits viral RNA-dependent RNA polymerase, blocking viral genome replication and transcription [84]. We identified nine RCTs that examined the efficacy of favipiravir in treating COVID-19. Five trials found significant differences between the favipiravir treatment and comparator groups [35, 45, 48, 52, 85] and four did not find significant differences [30, 40, 41, 44] (Table 3).

Zhao et al. conducted a multicentric open-label trial that compared favipiravir with a control group [45]. Patients were randomly assigned to receive favipiravir or treatments other than favipiravir, chosen at the discretion of the treating physician. Patients treated with favipiravir had a significantly shorter median time to positive-to-negative RT-PCR SARS-CoV-2 test conversion (17 days) compared to the control group (26 days; hazard ratio [HR]: 2.1 [95% confidence interval [CI] 1.1–4.0], p = 0.038). The trial ended after 30 days, at which time the favipiravir group had a significantly higher incidence of conversion to negative RT-PCR tests (80.6% [29/36]) compared to the control group (52.6% [10/19], p = 0.030). Mortality did not occur in either group within the 30-day study period.

Shinkai et al. investigated the efficacy of favipiravir in COVID-19 patients without oxygen therapy in a single-blind, placebo-controlled trial [52]. Patients received favipiravir or a placebo on the same schedule. They defined clinical improvement by four clinical parameters: temperature, oxygen saturation, chest imaging findings, and viral clearance assessed with RT-PCR. Patients treated with favipiravir met the criteria for clinical improvement significantly earlier (11.9 days [95% CI: 10.0–13.1 days]) than patients in the placebo group (14.7 days [95% CI: 10.5–17.9 days], p = 0.014). The difference in time to improvement was also significant in the covariate-adjusted Cox proportional hazards model (HR: 1.59 [95% CI 1.02–2.48]). Within the individual parameters, time to improvement of chest imaging findings (p = 0.029) and time to conversion to negative RT-PCR (p = 0.041) were significantly shorter in the favipiravir group compared to the placebo group, while temperature (p = 0.18) and SpO_2_ (p = 0.51) showed no significant difference (Table 3).

Udwadia et al. conducted a multicentric, open-label trial to compare favipiravir to standard supportive care alone [35]. No significant difference was found in time to conversion to negative RT-PCR tests (p = 0.1290) or duration of hospital stay (p = 0.1079). However, the favipiravir group had a significantly shorter time to resolution of clinical symptoms (3 days [95% CI: 3–4 days]) compared to the control group (5 days [95% CI: 4–6 days], p = 0.030).

Doi et al. [48] conducted a multicentric, open-label trial to compare patients treated with favipiravir starting on either day 1 (early) or day 6 (late) after their hospital admission. Patients received favipiravir for up to 10 days. Treatment could be discontinued after 6 days if their symptoms had resolved and they had two consecutive negative RT-PCR tests, meeting the requirements to be discharged from the hospital. Favipiravir did not significantly affect viral clearance by day 6 (HR: 1.416 [95% CI 0.764–2.623]). However, early treatment did lead to a significantly higher chance of viral clearance at day 6 in patients who were enrolled in the study more than three days after their first positive RT-PCR test (HR: 2.829 [95% CI 1.198–6.683]), indicating that there may be a window after infection where initiating treatment is more effective.

Chen et al. compared favipiravir with umifenovir in COVID-19 patients [85] in a multicentric, open-label trial. Umifenovir is an antiviral drug that prevents cell attachment and viral entrance by trimerization of the SARS-CoV-2 spike glycoprotein. This blockade forms a naked or immature virus that less contagious [86]. Patients also received standard therapy, which consisted of antivirals, steroids, traditional Chinese herbal medicines, immunomodulatory drugs, steroids, antibiotics, psychotic drugs, nutritional supplements, and oxygen support. The primary outcome was rate of clinical recovery at day 7. Secondary outcomes were all-cause mortality, dyspnea, respiratory failure, auxiliary oxygen therapy or noninvasive mechanical ventilation (NMV), latency to pyrexia and cough relief, and need for intensive care. While no differences were found in clinical recovery (favipiravir 61.2% [71/116]; umifenovir 51.7% [62/120]; *P* = 0.1396) or in most secondary outcomes between treatments, favipiravir did shorten the latency of pyrexia and cough relief.

Several trials did not find significant differences between treatment with favipiravir and their various comparator groups. Lou et al. conducted an open-label, single-center trial to evaluate the clinical outcomes and plasma concentrations of baloxavir acid and favipiravir in COVID-19 patients [30]. Patients were randomly assigned to one of three groups: a baloxavir marboxil group, a favipiravir group, and a control group, which included umifenovir. Median times from randomization to clinical improvement, viral negativity at day 7, and viral negativity at day 14 were similar between the three groups (Table 3). One patient in the baloxavir marboxil group and two patients in the favipiravir group were transferred to the ICU within 7 days due to declines in oxygen index or progressive disease on computed tomography (CT). One patient in the baloxavir marboxil group required extracorporeal membrane oxygenation (ECMO) support after 10 days.

Dabbous et al. conducted a multicentric trial comparing favipiravir and chloroquine (CQ) in patients with confirmed cases of COVID-19 [41]. There were no significant differences between the groups in mortality (p = 1.00), duration of hospital stay (p = 0.060), mechanical ventilation (p = 0.118), or oxygen saturation (p = 0.129). Bosaeed et al. also compared favipiravir (10 days) and HCQ [40]. Nearly half of the favipiravir group discontinued therapy before the end of the trial due to pill burden or personal preference. This study found no significant difference in conversion to negative RT-PCR tests (p = 0.73), time to clinical improvement (p = 0.29), duration of hospital stay (p = 0.42), 28-day mortality (p = 0.45), and 90-day mortality (p = 0.91). Solaymani-Dodaran et al. conducted a multicentric, open-label trial to compare favipiravir (in addition to HCQ) to LPV/r [44]. They found no significant differences between the groups for mortality (p = 0.52), transfer to the ICU (p = 0.47), time to clinical recovery (p = 0.54), incidence of clinical recovery (HR: 0.94 [95% CI 0.75–1.17]), or change in oxygen saturation (p = 0.46).

### Lopinavir/Ritonavir

LPV/r is an HIV-1 protease inhibitor combination. Ritonavir is combined with lopinavir to increase the latter’s plasma half-life by inhibiting cytochrome P450 [87]. LPV/r is approved by the FDA for treatment of HIV-1 infection in adult and pediatric patients [88]. LPV/r has also exhibited efficacy to treat influenza, severe acute respiratory syndrome (SARS), and Middle Eastern respiratory syndrome (MERS) infection [89–91]. Nine RCTs included LPV/r for COVID-19 therapy: two large trials (RECOVERY [26] and TOGETHER [27]), and seven relatively smaller trials (n = 86–664) [24, 31, 37, 40, 44, 47]. The trial conducted by Solaymani-Dodaran et al. compared LPV/r to favipiravir and found no significant differences, as discussed in the Favipiravir section above [44]. Similarly, none of the other trials identified a significant positive effect of LPV/r on outcomes in COVID-19 patients.

The RECOVERY trial was an open-label, platform trial conducted between March 19, 2020 and June 29, 2020 among 176 hospitals in the United Kingdom (UK). Patients were randomized to either standard of care alone or standard of care plus oral LPV/r for 10 days or until discharge. The primary outcome was 28-day all-cause mortality, which did not significantly differ between the intervention and control groups (rate ratio [RR] 1.03, 95% CI 0.91–1.17; *P* = 0.60), and the results were consistent among all pre-specified subgroups. There was also no difference in the time until discharge alive or proportion of patients discharged alive within 28 days (RR 0.98, 95% CI 0.91–1.05; *P* = 0.53). Additionally, there was no difference in the proportion of patients who met the composite endpoint of invasive mechanical ventilation or death among patients who were not on invasive mechanical ventilation at baseline (RR 1.09, 95% CI 0.99–1.20; *P* = 0.092).

The TOGETHER trial was conducted between June 2, 2020 and September 20, 2020 in Brazil [27]. The trial compared LPV/r to HCQ or placebo. The trial was discontinued early after finding no significant difference between the groups in COVID-19-associated hospitalization (LPV/r: HR, 1.16 [95% CI, 0.53–2.56]) or viral clearance at day 14 (LPV/r: odds ratio [OR], 1.04 [95% CI, 0.94–1.16]). Incidence of mortality was similar between the LPV/r and placebo groups. Ader et al. also compared LPV/r to HCQ and control, in addition to LPV/r with IFN-β-1a, and discontinued the LPV/r and HCQ arms early due to lack of significant difference in clinical status at day 15 compared to control [37]. Arabi et al. also conducted a randomized, multicentric trial comparing LPV/r, HCQ, or a combination to a control group with no antiviral therapy [40]. They found a 98.5% probability of harm compared to control for LPV/r alone based on in-hospital mortality.

Cao et al. conducted an open-label trial comparing LPV/r to standard of care in patients with SARS-CoV-2 infection and hypoxia [24]. There was no difference in time to clinical improvement (HR 1.24, 95% CI 0.90–1.72) or mortality at 28 days (19.2% vs. 25.0%; mean difference -5.8, 95% CI -17.3–5.7). The LPV/r group had a shorter median time to clinical improvement by one day compared to standard care alone on a modified intention-to-treat analysis (HR 1.39, 95% CI 1.00–1.91).

Three studies compared umifenovir to LPV/r [31, 47]. Li et al. conducted an exploratory trial to study the efficacy and safety of LPV/r versus umifenovir in patients with mild to moderate COVID-19 [31]. There were no differences in positive-to-negative conversion of SARS-CoV-2 RT-PCR tests on days 7 and 14. Also, there were no differences in mean time to test conversion (9.0, 9.1, and 9.3 days; *P* = 0.981) or in the conversion rate from moderate to severe/critical clinical status (23.5%, 8.6%, and 11.8%; *P* = 0.206) among LPV/r, umifenovir, and control groups, respectively.

Nojomi et al. investigated the efficacy of umifenovir compared to LPV/r in COVID-19 patients [31]. The patients were randomized to receive umifenovir or LPV/r for 7–14 days, based on disease severity, as well as HCQ on day 1. Patients that received umifenovir had a shorter duration of hospitalization (7.2 days) compared to patients that received LPV/r (9.6 days, *P* = 0.02). Moreover, 81% of patients in the umifenovir group had mild involvement on chest CT after 30 days of admission compared to 53% in the LPV/r group (*P* = 0.004).

Alavi Darazam et al. compared a combination of LPV/r, HCQ, and IFN-β1a with and without umifenovir in a single-center, open-label trial [47]. All patients received LPV/r, HCQ, and IFN-β1a. Half of the patients also received umifenovir. The groups did not have a significant difference in mortality (p = 0.62) or time to clinical improvement (p = 0.22), defined as improvement by two points on a seven-category ordinal scale. No significant difference in mortality was found between the groups when adjusted for time between symptom onset and trial enrollment either (presentation ≤ 7 days from symptom onset, p = 0.49; > 7 days, p = 1.00), indicating that starting treatment earlier is unlikely to affect the efficacy of combining umifenovir with LPV/r and other treatments.

### Remdesivir

Remdesivir is an RNA-dependent RNA polymerase inhibitor with in-vitro activity demonstrated against SARS-CoV-2 and MERS-CoV [34, 92]. It is FDA-approved for COVID-19 treatment in adult and pediatric patients (12 years or older and weighing at least 40 kg) requiring hospitalization [93]. We identified six trials used remdesivir to treat COVID-19. Three trials found significant differences between the remdesivir treatment and comparator groups [23, 34, 53] and three did not [36, 39, 51].

The Adaptive Covid-19 Treatment Trial (ACTT-1) was a multicentric, double-blind, placebo-controlled trial of remdesivir in patients with severe COVID-19 pneumonia [23]. Median recovery times were lower in the remdesivir group, with a rate ratio for recovery of 1.29 (95% CI 1.12–1.49, *P* < 0.001). The patients who received remdesivir were more likely to have clinical improvement by day 15 when compared to placebo (OR 1.5, 95% CI 1.2–1.9, after adjustment for actual disease severity). The Kaplan–Meier estimates of mortality at days 15 and 29 were 6.7% and 11.4% in the remdesivir group and 11.9% and 15.2% in the control group, respectively.

Spinner et al. compared remdesivir to standard of care in a multicentric, open-label trial of hospitalized patients with moderate COVID-19 pneumonia [34]. Patients were randomized to receive remdesivir for 5 or 10 days or standard care alone. On day 11, the odds for a better clinical status distribution were greater in the 5-day remdesivir group as compared to the standard care group (OR 1.65, 95% CI 1.09–2.48; *P* = 0.02) but was not significant between 10-day remdesivir and standard care groups (*P* = 0.18 by Wilcoxon Rank Sum test). Mortality at day 28 was 1%, 2%, and 2% in 5-day remdesivir, 10-day remdesivir, and standard care groups, respectively.

Goldman et al. also compared five- and ten-day courses of remdesivir [53]. Their open-label, phase 3 trial included patients with confirmed SARS-CoV-2 infection, SpO_2_ of ≤ 94% on room air, and radiologic evidence of pneumonia. The patients randomized to the 10-day group had significantly worse clinical status than those in the 5-day group, as assessed on a seven-category ordinal scale (p = 0.02). Discharge rates were higher in patients whose symptoms started less than 10 days before receiving the first dose of remdesivir (62%) than in those whose symptoms started 10 or more days before their first dose (49%), indicating that regardless of drug regimen, there may be advantages to starting remdesivir earlier.

Several trials found no significant effect of remdesivir on patient outcomes. Wang et al. conducted a double-blind, placebo-controlled, multicenter trial in COVID-19 patients with SpO_2_ ≤ 94% in room air or PaO_2_/FiO_2_ ratio ≤ 300 mmHg and radiological evidence of pneumonia [36]. Patients were assigned to remdesivir or placebo, along with standard of care. There was no difference in time to clinical improvement with remdesivir as compared to placebo (HR 1.23, 95% CI 0.87–1.75). Time to clinical improvement in a subgroup of patients with symptom duration ≤ 10 days was not significantly different with remdesivir compared to placebo (HR 1.52, 95% CI 0.95–2.43).

Mahajan et al. conducted a trial comparing remdesivir to standard of care in patients over 40 years old with moderate to severe COVID-19, but not on mechanical ventilation [51]. Clinical status was assessed with a six-point ordinal scale based on need for oxygen supplementation and ventilation, hospitalization and mortality status. The groups showed no significant difference in clinical status at day 24, including hospitalization and mortality (p = 0.749), despite the potential bias towards the remdesivir group found in the risk of bias assessment (Additional file 1). Discharge rates were higher for patients who received treatment less than 5 days after symptom onset regardless of treatment group. Barratt-Due et al. also conducted a RCT comparing remdesivir, HCQ, or standard of care alone and found no significant differences between the groups for in-hospital mortality (HR: 1.0 [95% CI 0.4–2.9]) and the groups had similar rates of viral clearance [39].

### Sofosbuvir/Daclatasvir

Sofosbuvir and daclatasvir are antiviral agents that inhibit viral RNA replication via NS5A and NS5B polymerase inhibition, respectively [94, 95]. Sofosbuvir and daclatasvir are FDA-approved for treatment of chronic hepatitis C [51]. SARS-CoV-2 possesses similar mechanisms of RNA replication as observed in other RNA viruses; as such, sofosbuvir and daclatasvir combined may demonstrate efficacy to inhibit SARS-CoV-2 replication [22, 96, 97]. We identified seven RCTs that used sofosbuvir and daclatasvir or a combination of sofosbuvir and other drugs to treat COVID-19. Of the RCTs that used sofosbuvir/daclatasvir, all five reported significantly better results for the treatment group for at least one outcome, although the magnitude of the effect was often small [22, 32, 33, 46, 49]. Of the three RCTs that included sofosbuvir combined with drugs other than daclatasvir, none reported significant differences between the treatment and control groups [28, 43, 46].

Sadeghi et al. conducted a phase 3, multicenter trial to compare the effects of sofosbuvir/daclatasvir with standard of care versus standard of care alone (HCQ and LPV/r at physician discretion) in moderate to severe COVID-19 patients [33]. Sofosbuvir/daclatasvir was started later than treatment in the control arm due to delays in receiving RT-PCR reports. Clinical recovery within 14 days from enrollment was achieved in 88% (29/33) of patients in the sofosbuvir/daclatasvir arm and 67% (22/33) of patients in the control arm (*P* = 0.076). Patients in the sofosbuvir/daclatasvir group experienced shorter hospital stays than patients in the control group (6 [4–8] days vs. 8 [5–13] days, respectively; *P* = 0.029), and the sofosbuvir/daclatasvir group exhibited a higher cumulative incidence of hospital discharge as compared to the control group (Gray’s *P* = 0.041). All-cause mortality was similar between groups.

Abbaspour Kasgari et al. conducted a single-center trial to evaluate the efficacy of sofosbuvir/daclatasvir in combination with ribavirin compared to standard of care (including other antivirals) for hospitalized patients with moderate COVID-19 [22]. Secondary outcomes included the frequency of ICU admission, duration of ICU admission, the frequency and time to recovery, mechanical ventilation, and invasive mechanical ventilation. There were no statistically significant differences in secondary outcomes between the two groups except for cumulative incidence of recovery (Gray’s *P* = 0.033), which was higher in the sofosbuvir/daclatasvir arm.

Roozbeh et al. investigated the efficacy of sofosbuvir/daclatasvir combined with HCQ for the treatment of COVID-19 outpatients compared to HCQ and standard of care using a double-blinded trial [32]. There was no difference between groups in the primary endpoint of symptom alleviation at day 7 follow-up or in the secondary endpoint of hospital admission (1 patient hospitalized in treatment group, 4 hospitalized in control group). Two patients in the sofosbuvir/daclatasvir arm reported fatigue at 1 month follow-up, while 16 patients reported fatigue in the control arm (*P* < 0.001). Dyspnea at 30-day follow-up was less common in the sofosbuvir/daclatasvir arm (14.8% [4/27]) than in the control arm (42.3% [11/26], *P* = 0.035).

El-Bendary et al. conducted a multi-centric trial comparing sofosbuvir/daclatasvir combined with HCQ to HCQ alone [49]. Patients treated with sofosbuvir/daclatasvir had a significantly lower median duration of hospitalization (8 days vs. 10 days in control group, p < 0.01) and a higher incidence of negative RT-PCR tests at day 14, with 84% (81/96) negative compared to 47% (37/78) negative in the control group (p < 0.01). The groups showed no significant differences in mortality (p = 0.07), ICU admission (p = 0.10), and clinical improvement on a seven-category ordinal scale (p = 0.07). The risk of bias assessment identified potential bias in favor of the sofosbuvir/daclatasvir group, but the potential bias was not expected to fully account for the effect observed (Additional file 1).

Abbass et al. compared sofosbuvir/daclatasvir to standard of care, with all patients receiving additional therapies, such as HCQ, ivermectin, LPV/r, or remdesivir, at the treating physician’s discretion [46]. Patients receiving sofosbuvir/daclatasvir showed significant clinical improvement compared to standard of care on both day 7 (p = 0.041) and day 10 (p = 0.040), as measured by the number of clinical symptoms experienced relative to day 3. The sofosbuvir/daclatasvir group also showed significant improvement in SpO_2_ (91.3% ± 4.7%) compared to the standard of care group (87.4% ± 8.8%, p = 0.016) starting on day 4 and continuing until the data collection ended on day 10. The groups did not have significant differences in incidence of viral clearance (p = 0.581), ICU admission (p = 0.254), or mortality (p = 0.329).

Three RCTs combined sofosbuvir with other drugs. Abbass et al. included sofosbuvir/ravidasvir along with sofosbuvir/daclatasvir [46]. They found no significant difference between sofosbuvir/ravidasvir and standard of care in clinical improvement (p = 0.66969), oxygen saturation (p = 0.054), viral clearance (p = 0.893), ICU admission (p = 0.254), or mortality at day 10 (p = 0.329). Khalili et al. compared sofosbuvir/ledipasvir to standard of care alone [28]. They found that sofosbuvir/ledipasvir had a shorter time to clinical improvement (2 [1–3.75]) compared to control (4 [2–5, p = 0.02), but no significant differences in incidence of clinical improvement (p = 0.65), duration of hospital stay (p = 0.98), or 14-day mortality (p = 0.60) between the groups. Sayad et al. compared sofosbuvir/velpatasvir to standard of care alone [43]. They likewise found no difference in 28-day mortality (p = 0.38), time to clinical improvement (HR: 1.2 [95% CI 0.6–2.2], p = 0.30), or conversion to negative RT-PCR tests (p = 0.49).

### Enisamium

One study evaluated the efficacy of enisamium, an antiviral drug whose metabolite is a viral RNA polymerase inhibitor [98]. Holubovska et al. conducted a double-blind, placebo-controlled, phase 3 trial comparing enisamium to a placebo [50]. No differences in time to recovery was found overall or among patients who did not initially require oxygen. However, among patients who did require oxygen supplementation when enrolled, enisamium decreased the recovery time (11.1 days) compared to the placebo group (13.9 days, p = 0.0259). All patients in the enisamium group recovered by day 21, while not all patients in the placebo group recovered before data collection for interim analysis ended on day 29.

## Discussion

Here, we examined the results of RCTs that investigated the efficacy of antiviral drugs for the treatment of COVID-19. While clinical trials of new antiviral candidates are ongoing, current evidence suggests that the success of antiviral therapy for COVID-19 treatment is dependent on multiple factors, including time from symptom onset to treatment.

Of the antiviral therapies we reviewed, the antiviral combination of sofosbuvir/daclatasvir most consistently exhibited efficacy for COVID-19 treatment across some clinical outcomes, although study sizes were small, and results were often inconsistent [22, 32, 33, 46, 49]. Inclusion criteria for COVID-19 severity varied between studies, which may account for some of the inconsistency. In the largest sofosbuvir RCT, consisting of 174 patients, El-Bendary et al. reported that patients treated with sofosbuvir/daclatasvir had a lower duration of hospitalization and higher incidence of viral clearance [49]. Other studies reported positive effects of sofosbuvir/daclatasvir, but which outcomes were reported varied [22, 33, 46]. However, Roozbeh et al. did not observe a difference in symptoms between groups with mild COVID-19 after 7 days of treatment [32], and there were no mortality benefits observed with sofosbuvir/daclatasvir treatment. Additionally, combinations of sofosbuvir with other drugs similar to daclatasvir did not lead to differences in outcomes compared to standard of care [28, 43, 46]. The fact that sofosbuvir/daclatasvir is available in pill form as opposed to IV (as is the case with remdesivir), its inexpensive price tag (14-day treatment is $4.42 USD) [99], and its favorable safety profile noted in hepatitis C treatment [100, 101] make sofosbuvir/daclatasvir an appealing option, provided its efficacy can be established in larger RCTs.

While remdesivir had shown early promise for effective treatment of COVID-19, the trials here demonstrated differing results. A previous meta-analysis found that remdesivir treatment of COVID-19 resulted in lower odds for mechanical ventilation or ECMO (OR 0.48, 95% CI 0.34, 0.69) and higher odds for hospital discharge at 28 days (OR 1.44, 95% CI 1.16, 1.79), while odds for mortality (OR 0.77, 95% CI 0.56, 1.06) were the same with or without remdesivir treatment [102]. Another meta-analysis found that remdesivir did not have a significant effect on the time to clinical improvement, or mortality but did have an effect on rate of recovered patients and hospital discharge [103]. Similarly, we found that four out of five studies comparing remdesivir to other treatments either failed to find significant differences in patient outcomes [36, 39, 51] or found unexpectedly opposing results between different remdesivir regimens and thus were inconclusive [34]. One placebo-controlled trial was stopped due to adverse events in patients treated with remdesivir [36]. Differences in findings may be due to different endpoints investigated or different levels of severity in patients, since the inclusion criteria varied between trials.

LPV/r and umifenovir were initially recommended for treatment of COVID-19 in China [33, 94]. Early observational and randomized controlled studies of LPV/r failed to find a benefit with treatment [104]. A small systematic review that examined the efficacy and safety of lopinavir/ritonavir in patients with COVID-19 found that lopinavir/ritonavir did not significantly affect death, viral clearance, or “radiological improvement” when compared to other interventions [105]. Subsequent results obtained from two RCTs, RECOVERY [26] and DISCOVERY [37], provided strong evidence against the use of LPV/r for COVID-19, and there were no benefits with early LPV/r treatment. Indeed, Arabi et al. reported that treatment with LPV/r led to worse outcomes compared to no antiviral treatment [40]. Thus, early administration of LPV/r or LPV/r use in patients with non-severe/non-critical forms of disease demonstrated little clinical value, and may be harmful.

The efficacy of umifenovir is unclear due to conflicting results obtained from relatively small studies. Of the four studies that included umifenovir in the study design [31, 47, 85], three studies failed to find a clinical benefit [31, 47, 85]. Moreover, early administration of umifenovir (median 6 days from symptom onset) did not influence the rate of positive-to-negative conversion of SARS-CoV-2 or rates of antipyresis, cough alleviation, or radiological findings of chest CT at days 7 or 14 after treatment [31]. In contrast, Nojomi et al. reported improvements in peripheral oxygen saturation, duration of hospitalization, need for ICU admission, white blood cell count, and erythrocyte sedimentation rate with umifenovir treatment as compared to LPV/r [31]. However, the time from symptom onset to treatment was not reported, and the group sizes were small (n = 50).

Similar to our study, Okoli et al. found that antivirals did not have an effect on either viral clearance or (all-cause mortality) but unlike our conclusions, they also found that antivirals did not significantly improve clinical progression [5]. Additionally, Lai, Chao, and Hsueh’s systematic review conclusions parallel ours as they found that remdesivir may increase time to clinical improvement and may be an effective treatment for mild and moderate COVID-19 and that sofosbuvir/daclatasvir may positively affect COVID-19 survival and clinical recovery [6]. However, their study does not include their methodology.

An important consideration when evaluating the efficacy of any drug, especially antivirals, is the state of disease course. Drugs that target viral replication, such as remdesivir, favipiravir, baloxavir marboxil, daclatasvir, and sofosbuvir, should be most effective if administered early in the viremic phase, as observed with other viruses (e.g. favipiravir treatment of Ebola) [106]. The SARS-CoV-2 viral load peaks within the first week of infection, which is earlier than that observed in SARS-CoV-1 (10–14 days) and MERS-CoV (7–10 days) [93]. Two of the trials we reviewed found that administering remdesivir within 10 days of symptom onset led to better patient recovery outcomes [23, 53]. Similarly, higher cumulative incidences of recovery were reported in moderate or severe COVID-19 patients treated with sofosbuvir/daclatasvir less than 8 days from symptom onset [22, 33]. In contrast, no differences in clinical outcomes were observed with baloxavir marboxil or favipiravir [30] or LPV/r when administered earlier relative to symptom onset. These data indicate that early administration of antiviral therapy may be critical to the efficacy of some COVID-19 treatments.

## Limitations

There were several limitations noted in the included studies. Standard of care varied across studies and included or could have included other antiviral therapies. In these cases, attributing a treatment effect to a specific drug can be difficult. Drugs that are not approved for use as antivirals may have unconfirmed antiviral activity. Additionally, there are a number of drugs that possess little effect individually but can elevate the overall antiviral benefit when administered with other antivirals (eg, ribavirin). Thus, the magnitude of treatment effect for a given antiviral drug is uncertain. Studies were not screened based on severity of cases included, which likely accounts for some of the inconsistency in results. Also, 36 non-English articles were excluded, which may impact the conclusions. Finally, nine studies had group sizes of 40 subjects or less [20, 22, 30, 32, 33, 43, 45, 46, 51], which may have resulted in insufficient statistical power and an increase in type II error (Additional file 2 and Additional file 3).

## Conclusions

The design and implementation of RCTs is a time-consuming process that struggles to keep pace with the needs of clinicians during a pandemic. However, the high level of evidence obtained through sufficiently powered RCTs can provide confidence and/or clarification regarding results obtained from various observational studies. For antivirals that exhibit efficacy for COVID-19 treatment, early administration may be a critical factor in determining the quality of outcome. Larger studies are needed for antivirals that are less-described in COVID-19 treatment, such as sofosbuvir/daclatasvir, as these drugs may have equal or superior clinical outcomes compared to current therapies and may be more amenable for widespread use (ie, cheaper costs, oral availability).

## Supplementary Information

**Additional file 1: Table S1. **Summary of risk of bias assessed with the Scottish Intercollegiate Guidelines Network (SIGN) randomized controlled trials checklist. Risk of bias assessment

**Additional file 2.** PRISMA checklist.

**Additional file 3.** PRISMA abstract checklist.

## Data Availability

The datasets generated and/or analyzed during the current study are available in the Nested Knowledge website [9].

## Abbreviations

ACTT-1: Adaptive Covid-19 Treatment Trial
CI: Confidence interval
COVID-19: Coronavirus disease 2019
CQ: Chloroquine
CT: Computed tomography
ECMO: Extracorporeal membrane oxygenation
GI: Gastrointestinal
HCQ: Hydroxychloroquine
HR: Hazard ratio
ICU: Intensive care unit
IFN: Interferon
IQR: Interquartile range
LPV/r: Lopinavir/ritonavir
MERS: Middle Eastern respiratory syndrome
NMV: Noninvasive mechanical ventilation
OR: Odds ratio
PRISMA: Preferred Reporting Items for Systematic Reviews and Meta-Analyses
RCTs: Randomized control trials
RR: Rate ratio
RT-PCR: Reverse transcription polymerase chain reaction tests
SaO_2_: Oxygen saturation
SARS: Severe acute respiratory syndrome
SARS-CoV-2: Severe acute respiratory syndrome coronavirus 2
SD: Standard deviation
SIGN: Scottish Intercollegiate Guidelines Network method
SpO_2_: Oxygen saturation
UK: United Kingdom
WBC: White blood cell count
