# Supplementary material for: Efficacy of antiviral therapies for COVID-19: a systematic review of randomized controlled trials
Source: BMC Infect Dis. 2022 Jan 31;22:107. doi: 10.1186/s12879-022-07068-0 (PMC8802260; doi:10.1186/s12879-022-07068-0)
Supplement: Supplementary file 1 — Additional file 1: Table S1. Summary of risk of bias assessed with the Scottish Intercollegiate Guidelines Network (SIGN) randomized controlled trials checklist. Risk of bias assessment [file 12879_2022_7068_MOESM1_ESM.docx]

**Additional Table 1. Summary of risk of bias assessed with the Scottish Intercollegiate Guidelines Network (SIGN) randomized controlled trials checklist**

| **Author** | **Study Title** | **How well was the study done to minimize bias?** | **If acceptable or low quality, what is the likely direction in which bias might affect the study results?** | **Taking into account clinical considerations, your evaluation of the methodology used, and the statistical power of the study, are you certain that the overall effect is due to the study intervention?** | **Level of evidence** |
| --- | --- | --- | --- | --- | --- |
| **Favipiravir** | | | | | |
| Bosaeed et al. [1] | Favipiravir and Hydroxychloroquine Combination Therapy in Patients with Moderate to Severe COVID- 19 (FACCT Trial): An Open-Label, Multicenter, Randomized, Controlled Trial | Acceptable (+) | Favors test treatment | Yes | 1+ |
| Chen et al. [2] | Favipiravir versus Arbidol for COVID-19: A Randomized Clinical Trial | Acceptable (+) | Neither favors test treatment nor reference treatment | Yes | 1+ |
| Dabbous et al. [3] | Efficacy of favipiravir in COVID-19 treatment: a multi-center randomized study | Acceptable (+) | Neither favors test treatment nor reference treatment | Yes | 1- |
| Doi et al. [4] | A Prospective, Randomized, Open-Label Trial of Early versus Late Favipiravir Therapy in Hospitalized Patients with COVID-19 | Acceptable (+) | Favors test treatment | Yes | 1+ |
| Lou et al. [5] | Clinical Outcomes and Plasma Concentrations of Baloxavir Marboxil and Favipiravir in COVID-19 Patients: An Exploratory Randomized,  Controlled Trial | Acceptable (+) | Favors test treatment | Yes | 1+ |
| Shinkai et al. [6] | Efficacy and Safety of Favipiravir in Moderate COVID- 19 Pneumonia Patients without Oxygen Therapy: A Randomized, Phase III Clinical Trial | Acceptable (+) | Favors test treatment | Yes | 1+ |
| Solaymani-Dodaran et al. [7] | Safety and efficacy of Favipiravir in moderate to severe  SARS-CoV-2 pneumonia | Acceptable (+) | Favors test treatment | Yes | 1+ |
| Udwadia et al. [8] | Efficacy and safety of favipiravir, an oral RNA-dependent RNA polymerase inhibitor, in mild-to-moderate COVID-19: A randomized, comparative, open-label, multicenter, phase 3 clinical trial. | Acceptable (+) | Favors test treatment | Yes | 1- |
| Zhao et al. [9] | Favipiravir in the treatment of patients with SARS-CoV-2 RNA recurrent positive after discharge: A multicenter, open-label, randomized trial | Acceptable (+) | Neither favors test treatment nor reference treatment | Yes | 1+ |
| **Lopinavir/Ritonavir** | | | | | |
| Ader et al. [10] | An open-label randomized, controlled trial of the effect of lopinavir/ritonavir, lopinavir/ ritonavir plus IFN-β-1a and hydroxychloroquine in hospitalized patients with COVID-19 | Acceptable (+) | Favors test treatment | Yes | 1+ |
| Alavi Darazam et al. [11] | Umifenovir in hospitalized moderate to severe COVID-19 patients: A  randomized clinical trial | Acceptable (+) | Favors test treatment | Yes | 1+ |
| Arabi et al. [12] | Lopinavir-ritonavir and hydroxychloroquine  for critically ill patients with COVID-19: REMAP-CAP randomized controlled trial | Acceptable (+) | Favors test treatment | Yes | 1+ |
| Cao et al. [13] | A Trial of Lopinavir–Ritonavir in Adults Hospitalized with Severe Covid-19 | Acceptable (+) | Favors test treatment | Yes | 1- |
| Li et al. [14] | Efficacy and safety of lopinavir/ritonavir or arbidol in adult patients with mild/moderate COVID-19: an exploratory randomized controlled trial | Acceptable (+) | Neither favors test treatment nor reference treatment | Yes | 1+ |
| Nojomi et al. [15] | Effect of Arbidol (Umifenovir) on COVID-19:  a randomized controlled trial | Acceptable (+) | Neither favors test treatment nor reference treatment | Yes | 1+ |
| RECOVERY collaborative group [16] | Lopinavir–ritonavir in patients admitted to hospital with COVID-19 (RECOVERY): a randomised, controlled, open-label, platform trial | Acceptable (+) | Favors test treatment | Yes | 1- |
| Reis et al. [17] | Effect of Early Treatment With Hydroxychloroquine or Lopinavir and Ritonavir on Risk of Hospitalization Among Patients With COVID-19 The TOGETHER Randomized Clinical Trial | High quality (++) | Not applicable | Yes | 1+ |
| **Remdesivir** | | | | | |
| Barratt-Due et al. [18] | Evaluation of the Effects of Remdesivir and Hydroxychloroquine on Viral Clearance in COVID-19: A Randomized Trial | Acceptable (+) | Favors test treatment | Yes | 1+ |
| Beigel et al. [19] | Remdesivir for the Treatment of Covid-19 - Final Report | High quality (++) | Not applicable | Yes | 1++ |
| Goldman et al. [20] | Remdesivir for 5 or 10 Days in Patients  with Severe Covid-19 | Acceptable (+) | Favors test treatment | Yes | 1- |
| Mahajan et al. [21] | Clinical outcomes of using remdesivir in patients with moderate to severe COVID-19: A prospective randomised study | Low quality (-) | Favors test treatment | Yes | 1- |
| Spinner et al. [22] | Effects of Remdesivir vs Standard Care on Clinical Status at 11 Days in Patients With Moderate COVID-19 A Randomized Clinical Trial | High quality (++) | Not applicable | Yes | 1++ |
| Wang et al. [23] | Remdesivir in adults with severe COVID-19: a randomised, double-blind, placebo-controlled, multicentre trial | High quality (++) | Not applicable | Yes | 1++ |
| **Sofosbuvir** | | | | | |
| Abbaspour-Kasgari et al. [24] | Evaluation of the efficacy of sofosbuvir plus daclatasvir in combination with ribavirin for hospitalized COVID-19 patients with moderate disease compared with standard care: a single-centre, randomized controlled trial | Acceptable (+) | Favors test treatment | Yes | 1- |
| Abbass et al. [25] | Efficacy and safety of sofosbuvir plus daclatasvir or ravidasvir in patients with COVID‐19: A randomized controlled trial | Acceptable (+) | Favors test treatment | Yes | 1+ |
| El-Bendary et al. [26] | Efficacy of combined Sofosbuvir and Daclatasvir in the treatment of COVID-19 patients with pneumonia: a multicenter Egyptian study | Low quality (-) | Favors test treatment | Yes | 1- |
| Khalili et al. [27] | Efficacy and safety of sofosbuvir/ledipasvir in treatment of  patients with COVID-19; A randomized clinical trial | Acceptable (+) | Favors test treatment | Yes | 1+ |
| Sadeghi et al. [28] | Sofosbuvir and daclatasvir compared with SoC in the treatment of patients admitted to hospital with moderate or severe coronavirus infection (COVID-19): a randomized controlled trial | Acceptable (+) | Favors test treatment | Yes | 1+ |
| Sayad et al. [29] | Efficacy and safety of sofosbuvir/velpatasvir versus the standard of care in adults hospitalized with COVID-19: a single-centre, randomized controlled trial | Acceptable (+) | Favors test treatment | Yes | 1+ |
| Roozbeh et al. [30] | Sofosbuvir and daclatasvir for the treatment of COVID-19 outpatients: a double-blind, randomized controlled trial | High quality (++) | Not applicable | Yes | 1++ |
| **Enisamium** | | | | | |
| Holubovska1 et al. [31] | Enisamium is an inhibitor of the SARS-CoV-2 RNA polymerase and shows improvement of recovery in COVID-19 patients in an interim analysis of a clinical trial | Acceptable (+) | Neither favors test treatment nor reference treatment | Yes |  |

Data are presented as mean ± standard deviation, median (IQR), or n (%) unless otherwise stated.

* statistically different from comparator

AIDS=autoimmune deficiency syndrome; ALT= alanine aminotransferase; ARB=Arbidol; AST= aspartate aminotransferase; B/M=baloxavir/marboxil; BMI=body mass index; CKD=chronic kidney disease; CQ; chloroquine; CT=computed tomography; D#=day #; DB=double-blind; D/C=darunavir/cobicistat; ECG=electrocardiogram; ECMO=extracorporeal membrane oxygenation; eGFR=estimated glomerular filtration rate; FVP=favipiravir; HAART=highly active antiretroviral therapy; HCQ=hydroxychloroquine; HCV=hepatitis C virus; HIV=human immunodeficiency virus; IFN=interferon; IU=international units; LPV/r=lopinavir/ritonavir; OL=open-label; PaO2/FiO2=arterial partial pressure of oxygen/fraction of inspired oxygen ratio; QTc=corrected QT interval; RDV=Remdesivir; RCT=randomized controlled trial; RT-PCR=reverse transcriptase polymerase chain reaction; SaO2=arterial oxygen saturation; SoC=standard of care; SOF/LDP= Sofosbuvir/ledipasvir; SOF/VEL=sofosbuvir/velpatasvir; SpO2=oxygen saturation; Sx=symptom; Tx=treatment; ULN=upper limit of normal; WBC=white blood cells

**References**

[1] Bosaeed M, Mahmoud E, Alharbi A, Altayib H, Albayat H, Alharbi F, et al. Favipiravir and Hydroxychloroquine Combination Therapy in Patients with Moderate to Severe COVID-19 (FACCT Trial): An Open-Label, Multicenter, Randomized, Controlled Trial. Infect Dis Ther 2021:1-17.

[2] Chen C, Zhang Y, Huang J, Yin P, Cheng Z, Wu J, et al. Favipiravir versus Arbidol for COVID-19: A Randomized Clinical Trial. MedRxiv 2020.

[3] Dabbous HM, Abd-Elsalam S, El-Sayed MH, Sherief AF, Ebeid FFS, El Ghafar MSA, et al. Efficacy of favipiravir in COVID-19 treatment: a multi-center randomized study. Arch Virol 2021;166(3):949-54.

[4] Doi Y, Hibino M, Hase R, Yamamoto M, Kasamatsu Y, Hirose M, et al. A Prospective, Randomized, Open-Label Trial of Early versus Late Favipiravir Therapy in Hospitalized Patients with COVID-19. Antimicrobial agents and chemotherapy 2020;64(12).

[5] Lou Y, Liu L, Yao H, Hu X, Su J, Xu K, et al. Clinical Outcomes and Plasma Concentrations of Baloxavir Marboxil and Favipiravir in COVID-19 Patients: An Exploratory Randomized, Controlled Trial. Eur J Pharm Sci 2021;157:105631.

[6] Shinkai M, Tsushima K, Tanaka S, Hagiwara E, Tarumoto N, Kawada I, et al. Efficacy and Safety of Favipiravir in Moderate COVID-19 Pneumonia Patients without Oxygen Therapy: A Randomized, Phase III Clinical Trial. Infect Dis Ther 2021:1-21.

[7] Solaymani-Dodaran M, Ghanei M, Bagheri M, Qazvini A, Vahedi E, Hassan Saadat S, et al. Safety and efficacy of Favipiravir in moderate to severe SARS-CoV-2 pneumonia. Int Immunopharmacol 2021;95:107522.

[8] Udwadia ZF, Singh P, Barkate H, Patil S, Rangwala S, Pendse A, et al. Efficacy and safety of favipiravir, an oral RNA-dependent RNA polymerase inhibitor, in mild-to-moderate COVID-19: A randomized, comparative, open-label, multicenter, phase 3 clinical trial. Int J Infect Dis 2021;103:62-71.

[9] Zhao H, Zhang C, Zhu Q, Chen X, Chen G, Sun W, et al. Favipiravir in the treatment of patients with SARS-CoV-2 RNA recurrent positive after discharge: A multicenter, open-label, randomized trial. Int Immunopharmacol 2021;97:107702.

[10] Ader F, Peiffer-Smadja N, Poissy J, Bouscambert-Duchamp M, Belhadi D, Diallo A, et al. An open-label randomized controlled trial of the effect of lopinavir/ritonavir, lopinavir/ritonavir plus IFN-β-1a and hydroxychloroquine in hospitalized patients with COVID-19. Clin Microbiol Infect 2021.

[11] Alavi Darazam I, Shokouhi S, Mardani M, Pourhoseingholi MA, Rabiei MM, Hatami F, et al. Umifenovir in hospitalized moderate to severe COVID-19 patients: A randomized clinical trial. Int Immunopharmacol 2021;99:107969.

[12] Arabi YM, Gordon AC, Derde LPG, Nichol AD, Murthy S, Beidh FA, et al. Lopinavir-ritonavir and hydroxychloroquine for critically ill patients with COVID-19: REMAP-CAP randomized controlled trial. Intensive Care Med 2021;47(8):867-86.

[13] Cao B, Wang Y, Wen D, Liu W, Wang J, Fan G, et al. A Trial of Lopinavir-Ritonavir in Adults Hospitalized with Severe Covid-19. N Engl J Med 2020;382(19):1787-99.

[14] Li Y, Xie Z, Lin W, Cai W, Wen C, Guan Y, et al. Efficacy and Safety of Lopinavir/Ritonavir or Arbidol in Adult Patients with Mild/Moderate COVID-19: An Exploratory Randomized Controlled Trial. Med (N Y) 2020;1(1):105-13.e4.

[15] Nojomi M, Yassin Z, Keyvani H, Makiani MJ, Roham M, Laali A, et al. Effect of Arbidol (Umifenovir) on COVID-19: a randomized controlled trial. BMC Infect Dis 2020;20(1):954.

[16] Group RC. Lopinavir-ritonavir in patients admitted to hospital with COVID-19 (RECOVERY): a randomised, controlled, open-label, platform trial. Lancet 2020.

[17] Reis G, Moreira Silva E, Medeiros Silva DC, Thabane L, Singh G, Park JJH, et al. Effect of Early Treatment With Hydroxychloroquine or Lopinavir and Ritonavir on Risk of Hospitalization Among Patients With COVID-19: The TOGETHER Randomized Clinical Trial. JAMA Netw Open 2021;4(4):e216468.

[18] Barratt-Due A, Olsen IC, Nezvalova-Henriksen K, Kåsine T, Lund-Johansen F, Hoel H, et al. Evaluation of the Effects of Remdesivir and Hydroxychloroquine on Viral Clearance in COVID-19 : A Randomized Trial. Ann Intern Med 2021.

[19] Beigel JH, Tomashek KM, Dodd LE, Mehta AK, Zingman BS, Kalil AC, et al. Remdesivir for the Treatment of Covid-19 - Final Report. N Engl J Med 2020;383(19):1813-26.

[20] Goldman JD, Lye DCB, Hui DS, Marks KM, Bruno R, Montejano R, et al. Remdesivir for 5 or 10 Days in Patients with Severe Covid-19. N Engl J Med 2020;383(19):1827-37.

[21] Mahajan L, Singh AP, Gifty. Clinical outcomes of using remdesivir in patients with moderate to severe COVID-19: A prospective randomised study. Indian journal of anaesthesia 2021;65(Suppl 1):S41-s6.

[22] Spinner CD, Gottlieb RL, Criner GJ, Arribas Lopez JR, Cattelan AM, Soriano Viladomiu A, et al. Effect of Remdesivir vs Standard Care on Clinical Status at 11 Days in Patients With Moderate COVID-19: A Randomized Clinical Trial. JAMA 2020;324(11):1048-57.

[23] Wang Y, Zhang D, Du G, Du R, Zhao J, Jin Y, et al. Remdesivir in adults with severe COVID-19: a randomised, double-blind, placebo-controlled, multicentre trial. Lancet 2020;395(10236):1569-78.

[24] Abbaspour Kasgari H, Moradi S, Shabani AM, Babamahmoodi F, Davoudi Badabi AR, Davoudi L, et al. Evaluation of the efficacy of sofosbuvir plus daclatasvir in combination with ribavirin for hospitalized COVID-19 patients with moderate disease compared with standard care: a single-centre, randomized controlled trial. J Antimicrob Chemother 2020;75(11):3373-8.

[25] Abbass S, Kamal E, Salama M, Salman T, Sabry A, Abdel-Razek W, et al. Efficacy and safety of sofosbuvir plus daclatasvir or ravidasvir in patients with COVID-19: A randomized controlled trial. Journal of medical virology 2021.

[26] El-Bendary M, Abd-Elsalam S, Elbaz T, El-Akel W, Cordie A, Elhadidy T, et al. Efficacy of combined Sofosbuvir and Daclatasvir in the treatment of COVID-19 patients with pneumonia: a multicenter Egyptian study. Expert Rev Anti Infect Ther 2021:1-5.

[27] Khalili H, Nourian A, Ahmadinejad Z, Emadi Kouchak H, Jafari S, Dehghan Manshadi SA, et al. Efficacy and safety of sofosbuvir/ ledipasvir in treatment of patients with COVID-19; A randomized clinical trial. Acta Biomed 2020;91(4):e2020102.

[28] Sadeghi A, Ali Asgari A, Norouzi A, Kheiri Z, Anushirvani A, Montazeri M, et al. Sofosbuvir and daclatasvir compared with standard of care in the treatment of patients admitted to hospital with moderate or severe coronavirus infection (COVID-19): a randomized controlled trial. J Antimicrob Chemother 2020;75(11):3379-85.

[29] Sayad B, Khodarahmi R, Najafi F, Miladi R, Mohseni Afshar Z, Mansouri F, et al. Efficacy and safety of sofosbuvir/velpatasvir versus the standard of care in adults hospitalized with COVID-19: a single-centre, randomized controlled trial. J Antimicrob Chemother 2021;76(8):2158-67.

[30] Roozbeh F, Saeedi M, Alizadeh-Navaei R, Hedayatizadeh-Omran A, Merat S, Wentzel H, et al. Sofosbuvir and daclatasvir for the treatment of COVID-19 outpatients: a double-blind, randomized controlled trial. J Antimicrob Chemother 2021;76(3):753-7.

[31] Holubovska O, Bojkova D, Elli S, Bechtel M, Boltz D, Muzzio M, et al. Enisamium is an inhibitor of the SARS-CoV-2 RNA polymerase and shows improvement of recovery in COVID-19 patients in an interim analysis of a clinical trial. medRxiv 2021.
